# Synthetic Tuning of Exciton–Phonon Coupling in Janus WS_2(1‑*x*)_Se_2*x*_ Monolayers Revealed by Resonant Raman Excitation Spectroscopy for Optoelectronic Applications

**DOI:** 10.1021/acsanm.6c01104

**Published:** 2026-06-17

**Authors:** Alexander A. Puretzky, Liangbo Liang, Sumner B. Harris, Yu-Chuan Lin, David B. Geohegan

**Affiliations:** † Center for Nanophase Materials Sciences, 6146 Oak Ridge National Laboratory, Oak Ridge, Tennessee 37831, United States; ‡ Department of Materials Science and Engineering, 34914 The Pennsylvania State University, University Park, Pennsylvania 16802, United States; § Department of Materials Science and Engineering, 122608 University of Tennessee, Knoxville, Tennessee 37996, United States

**Keywords:** Raman excitation profiles, Janus monolayers, pulsed laser ablation, exciton−phonon interaction, *in situ* diagnostics

## Abstract

Janus monolayers, such as WSSe, have broken out-of-plane symmetry and an intrinsic dipole moment, impacting exciton transport, lifetime, and phonon interactions while imbuing piezoelectric, photocatalytic, and Rashba spin-splitting properties to transition metal dichalcogenides (TMDs). The new properties of this atomically thin material can be used for optoelectronic device applications. As TMDs are converted into Janus monolayers, e.g., top selenization of WS_2_ to WSSe, the bandgap and structure smoothly evolve, impacting not only the formation of excitons but also their complex interactions with different phonon modes. Resonant Raman excitation profiles (REPs) are uniquely well-suited to reveal both excitonic transitions and exciton–phonon coupling. Here, the resonant REPs of $A_{1}^{\prime }$ WS_2_ and *A*_1_ WSSe modes are measured to understand the strength of their coupling with the A, B, and C excitonic bands of a WS_2_ monolayer throughout its stepwise transformation into Janus WSSe by pulsed laser deposition (PLD) of energetic selenium species. *In situ* Raman spectroscopy during deposition is used to controllably prepare stable intermediate Janus structures, WS_2(1‑*x*)_Se_2*x*_ (0 ≤ *x* ≤ 0.5), for *ex situ* measurement of their resonant REPs. As *x* increases, REPs reveal not only pronounced excitonic bands that gradually shift toward lower photon energies but also strong, mode-selective exciton–phonon coupling. First-principles resonant Raman simulations independently predict this spectral behavior and are shown capable of matching the spectrally broadened, experimentally observed REP profiles in this model system, indicating their strong predictive capability for future experiments. The combination of controlled synthesis, REP characterization, and predictive theory employed here demonstrates a powerful pathway to understand and ultimately tune exciton–phonon interactions for future quantum optical devices.

## Graphical abstract

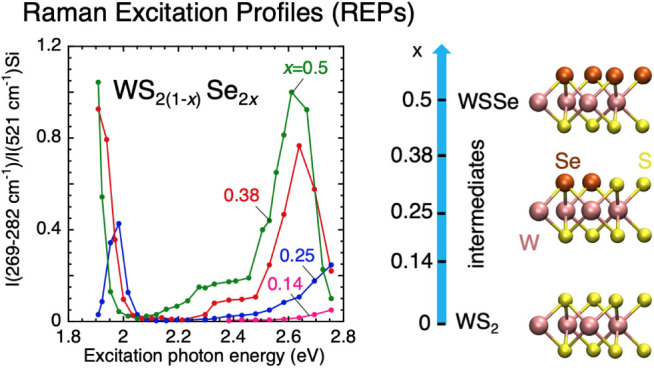

## Introduction

Atomically thin two-dimensional (2D) materials have been extensively studied owing to their diverse fundamental physical behaviors and technologically relevant properties.^1,2^ Transition-metal dichalcogenides (TMDs), such as MoS_2_, WS_2_, and WSe_2_, are among the most interesting classes of 2D materials with great application potential such as flexible electronics, photodetectors, energy storage devices, and electrocatalysts.^3–5^ They possess the X-M-X sandwich structure, with M as the transition-metal atom and X as the chalcogen atoms. Furthermore, by replacing one chalcogen layer with a different chalcogen atom, an asymmetric X-M-Y structure can be realized with new functionalities that are not present in the parent structures. For instance, a built-in electric dipole moment emerges, which can lead to strong piezoelectricity, along with the Rashba effect arising from the broken inversion symmetry and large spin–orbit coupling.^4,6^ Therefore, the synthesis and characterization of Janus TMDs have been gaining momentum in the field,^7,8^ which are also driven by the important optoelectronic applications of Janus 2D nanomaterials that include photodetectors and solar cells, as discussed in detail in a recent comprehensive review.^9^

Additional flexibility in the optoelectronic properties of these new materials comes from the possibility to synthesize intermediate Janus alloy structures with the composition MX_2(1‑*x*)_Y_2*x*_ (0 ≤ *x* ≤ 1), where X and Y are dissimilar chalcogen atoms. This is similar to widely studied TMD alloys^10,11^ when dissimilar chalcogen atoms are randomly distributed on both sides of a TMD monolayer, which results in a tunable electronic band structure^12,13^ that is important for optoelectronic applications, such as solar cells and photodetectors. Recently, the effects of defects on the optoelectronic and vibrational properties of Janus MoS_2(1–*x*)_Se_2*x*_ alloys have also been studied.^14^

However, a systematic and in-depth study of the electronic, optical, and vibrational properties of the Janus 2D materials, and especially their intermediate structures, is still lacking. To facilitate the development of Janus TMDs, it is highly desirable to have a rapid, simple, and *in situ* characterization technique to monitor the conversion process from the parent structure to the Janus and to study their electronic, optical, and vibrational properties simultaneously.

Raman spectroscopy stands out as such an enabling method. It gives structural information based on measurements of inelastically scattered light by phonons and is routinely used for the characterization of 2D materials.^15–19^ Commonly, these measurements utilize a single or a few laser excitation wavelengths to acquire Raman spectra. However, continuously tuning the excitation wavelength is beneficial not only for improving the Raman scattering efficiency through resonant excitation but also for studying excitonic states and their interactions with phonons. Intensity variations of a specific phonon mode measured during the tuning of excitation wavelengths give a Raman excitation profile (REP) that contains information about the exciton band positions, lifetimes, and the strength of the exciton–phonon couplings. In addition to the identification of excitonic transitions, which can also be accomplished by common UV–vis spectroscopy, REPs provide mode-selective excitation, allowing the avoidance of multiple overlapping transitions that result in broad UV–vis absorption bands.

Although the importance of the REP measurements and their model-based fittings has been understood since the early days of 2D material studies,^20^ this type of measurement is not common in the 2D material field, mainly due to the relative experimental complexity that, in an ideal case, requires a triple monochromator, a wavelength-tunable continuous wave (CW) laser with sufficient tunability to excite the excitonic bands of a 2D crystal, and normalization of Raman mode intensities for spectral REP corrections. Scheuschner et al. have measured REPs of the $A_{1}^{\prime }\left(A_{1g}\right)$ mode of exfoliated MoS_2_ mono-, bi-, and trilayers in the energy range of the A and B excitons.^20^ Later, Carvalho et al. investigated the REPs of $A_{1}^{\prime }$ and *E*′ modes in a monolayer and in few layers of MoS_2_ with the conclusions that $A_{1}^{\prime }\left(A_{1g}\right)$ mode interacts with the A and B excitons, but not with the C exciton,^21^ but later recalled this claim and concluded that their corrected REPs showed that excitation in all A, B, and C excitonic bands resulted in enhancement of the *A*- and *E*-symmetry Raman modes.^22^ REPs of other 2D materials, such as MoSe_2_,^23–25^ ReS_2_,^26^ WS_2_, and WSe_2_,^27–30^ have also been studied. For example, McDonnell et al., revealed contributions of exciton–exciton, trion–trion, and exciton–trion scattering to REPs by tuning excitation in the range of photon energies of the A exciton of a WS_2_ monolayer.^30^ However, REPs of Janus 2D materials have not been studied yet.

In our previous work, we used pulsed laser ablation of a Se target to generate kinetic energy-controlled fluxes of Se in order to controllably transform WS_2_ monolayer crystals into Janus WSSe.^7,8^ This approach makes possible the selective replacement of the topmost sulfur atoms in a WS_2_ layer to a complete Janus WSSe monolayer through transient fractional Janus intermediates, WS_2(1–*x*)_ Se_2*x*_, where *x* is the conversion fraction.^8^ Using 532 nm (2.33 eV) excitation, the Raman mode intensities and shifts were monitored *in situ* during the PLD conversion and compared with *ex situ* atomic characterization and our theoretical calculations to reveal the transformation kinetics.^8^

Here, we use a tunable laser to measure REPs of the $A_{1}^{\prime }\left({\mathrm{WS}}_{2}\right)$ and *A*_1_(WSSe) modes while converting WS_2_ monolayers to Janus WSSe via pulsed laser ablation of a Se target, arresting stable fractional intermediates WS_2(1–*x*)_Se_2*x*_ (0 ≤ *x* ≤ 0.5). The intermediates exhibit pronounced A, B, and C exciton bands that red-shift smoothly with *x*. First-principles resonant-Raman simulations reproduce the measured profiles and confirm strong, mode-selective exciton–phonon coupling. These results allow us to track A, B, and C excitons across stable Janus intermediates rather than only their parent structures, establishing tunable-laser REP as a nondestructive, quantitative pathway for deterministic bandgap and electronic-state engineering in Janus/alloyed TMDC monolayers. The ability to controllably synthesize alloy 2D materials by pulsed laser deposition and probe exciton–phonon coupling by tunable Raman excitation is important for applications in future quantum optical devices.

## Experimental Section

### Synthesis of Janus WSSe Monolayers and Their Intermediates

The WS_2_ monolayer crystals were synthesized on a c-plane sapphire substrate by metal–organic chemical vapor deposition (MOCVD) and were transferred to a SiO_2_ (300 nm)/Si (100) substrate for further conversion by PLD to Janus structures and their intermediate alloys. Intermediate Janus structures WS_2(1‑*x*__)_Se_2*x*_ were synthesized by conversion of WS_2_ monolayers by pulsed laser ablation of a Se target (Figure 1). The synthesis method is the same as the one described in ref.^8^. Briefly, the maximum kinetic energy of the Se plasma plume was controlled to 3.2 eV/Se atom by ablating the Se target with a KrF laser (248 nm, 1.0 J cm^–2^, spot area 0.041 cm^2^, 10 cm target-sample distance, 5 Hz repetition rate) into a 14 mTorr Ar background gas. The WS_2_ sample was held at 450 °C to evaporate the excess Se deposited per laser pulse. To obtain the range of intermediate alloy compositions, real-time measurement of the WS_2(1‑*x*)_ Se_2*x*_ *A*_1_ Raman peak positions with 532 nm laser excitation was used during the conversion process as feedback to control the implantation dose (pulse number). X-ray photoelectron spectroscopy was used to confirm the composition and is presented in ref.^8^.

**Figure 1:**
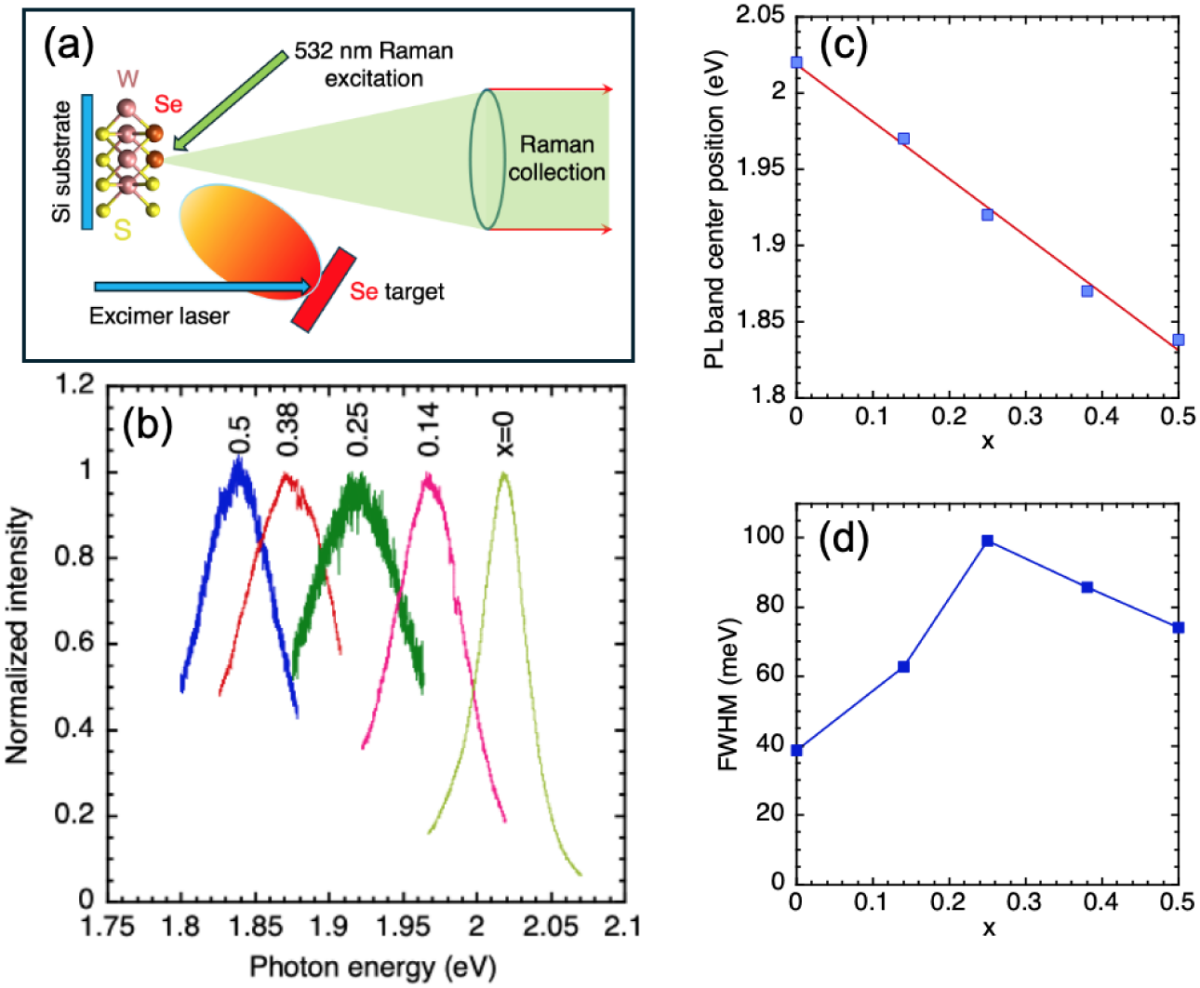
PLD synthesis of Janus structures with tunable bandgap. (a) Schematic of the experimental setup for the controllable synthesis of Janus monolayers and their intermediate structures with real-time Raman feedback monitoring of the growth process. An SiO_2_/Si substrate with a continuous monolayer of WS_2_ is exposed to a plasma plume from a laser-ablated Se target (KrF laser, 248 nm, 1.0 J cm^–2^, spot area 0.041 cm^2^, 10 cm target-sample distance, 5 Hz repetition rate) in a 14 mTorr Ar background gas. The number of laser pulses needed to achieve a specific intermediate Janus structure, *x*, is controlled by feedback from *in situ* Raman scattering under 532 nm excitation using the WS_2(1‑*x*__)_ Se_2*x*_ *A*_1_ mode center position. (b) PL bands of WS_2(1–*x*)_ Se_2*x*_ measured at different *x,* as indicated at the top. (c) The PL band center positions and (d) full width at half-maximum (fwhm) versus *x*. The solid squares show the experimental points, and the line in (c) is a linear fit with a slope of −0.37 eV/*x*.

### Raman Measurements

Excitation-tunable Raman measurements were performed using a triple spectrometer (Jobin-Yvon T64000) equipped with three 1800 grooves/mm gratings and a CCD detector (Synapse Plus, Horiba). All measurements were performed under a microscope in a backscattering configuration. A tunable (450–525 nm and 540–650 nm) visible CW optical parametric oscillator (OPO) (C-Wave, Hubner Photonics) was used as a Raman excitation source to measure REPs of Janus WSSe monolayers and their intermediates. In addition, one point in the gap of the OPO (525–540 nm) at 532 nm was measured using a 532 nm laser (Excelsior, Spectra-Physics). Excitation laser light was focused onto the sample with a 100× objective (numerical aperture NA = 0.9) to a spot size of about 1 μm. The excitation laser power on the sample was maintained at around ∼1 mW across all used wavelengths. All Raman spectra were measured in collinear polarization geometry, with special attention given to the SiO_2_ (300 nm)/Si (100) substrate orientation relative to the incoming laser polarization, which is important since all the measured Raman peaks were normalized to the Si peak at 521 cm^–1^.

### Photoluminescence Measurements

The PL spectra were measured using a custom-built micro-PL setup. The PL was excited with a CW diode-pumped solid-state laser (Excelsior, Spectra Physics, 532 nm, 100 mW) through an upright microscope using a 100× working distance objective with a numerical aperture (NA) = 0.9 (beam spot ∼ 1 μm). The typical incident laser power on the sample was maintained at approximately 5 μW to reduce possible laser heating and damage to the samples during PL spectra acquisition. The PL light was analyzed by a spectrometer (Spectra Pro 2300i, Acton, f = 0.3 m) with a 150 grooves/mm grating and a CCD camera (Pixis 256BR, Princeton Instruments).

## Computational Details

Plane-wave density functional theory (DFT) calculations were performed using the VASP package equipped with the projector-augmented-wave (PAW) method for electron–ion interactions (see Note S1 in the Supporting Information SI for details).^31^ The exchange-correlation functional within the local density approximation (LDA) was adopted to calculate phonon properties. For monolayer WS_2_ and WSSe, they were simulated by a periodic slab geometry with a vacuum separation distance of 21 Å in the *z*-direction to avoid spurious interactions with periodic neighboring unit cells. Both the atomic positions and in-plane lattice constants were optimized until the residual forces were below 0.001 eV/Å. The energy cutoff was 400 eV and the k-point sampling was 24 × 24 × 1. Then, the dynamic matrix was calculated using the finite difference scheme implemented in the Phonopy software.^32^ Hellmann–Feynman forces in the 4 × 4 × 1 supercell were computed by VASP for both positive and negative atomic displacements (Δ = 0.03 Å) and then used in Phonopy to construct the dynamic matrix, whose diagonalization provides phonon frequencies and eigenvectors.

Raman intensities were calculated within the Placzek approximation.^33,34^ For the *j*-th phonon mode, the Raman intensity is *I* ∝ | ***e***_i_·*R̃*· $e_{s}^{T}$ |^2^, where ***e***_i_ and ***e***_s_ are the electric polarization vectors of the incident and scattered light, respectively, and *R̃* is the Raman tensor of the phonon mode. The matrix element of the (3 × 3) Raman tensor *R̃* of the *j*-th phonon mode is related to the derivative of the dielectric tensor over the Cartesian atomic displacement, which is then multiplied by the phonon eigendisplacements of the *j*-th mode to be equivalent to the derivative of the dielectric tensor over the phonon normal-mode coordinate (more details in Note S1 in the SI).^33–38^ To capture the dependence of Raman intensities on the laser excitation energy, we need to compute the dynamic dielectric tensors *ε*_*αβ*_ as a function of the laser excitation energy.^39–44^ To accurately do that, many-body perturbation theory within the *GW* approximation (*G* is the Green’s function; *W* is the Coulomb interaction) is needed to obtain accurate electronic eigenvalues, including unoccupied states, and solving the Bethe–Salpeter equation (BSE) is required to describe optical excitations and excitonic effects.^45–48^ This would allow us to capture many-body electron–electron and electron–hole interactions in the calculated Raman spectra as well as REPs (note S1 in the SI). Based on the phonon frequencies, phonon eigenvectors, and the derivatives of dielectric tensors, the Raman tensor *R̃* and Raman intensity of any phonon mode at any laser excitation energy can be obtained using an open-source Python package, “SpectroPy” developed by us (available at https://github.com/TheorySpectroPy/SpectroPy), thereby yielding REPs.

We note that the Placzek approximation has been frequently used in the resonance region in solid-state Raman simulations and often gives qualitatively or semiquantitatively correct intensity profiles, despite its inherent limitations.^28,39,40,42,43,49^ According to Miranda et al.,^49^ it is valid under the condition ℏω_μ_ ≪ |ℏω – Δ*E* + iγ |, where ω_μ_ is the frequency of phonon mode μ, ℏω is the photon energy of the incident laser light, Δ*E* is the energy of an electronic transition, and γ is the broadening related to the lifetime of the electronic excitation. In the resonant regime, where the laser photon energy matches the energy of an electronic transition (ℏ*ω* = Δ*E*), the condition can still be fulfilled for phonon modes with energies ω_μ_ smaller than the electronic broadening γ. The first-order Raman modes we studied in TMDs, without multiple vibrational excitations involved, are around 418 and 282 cm^–1^ and also shift to lower energies in the intermediate Janus structures, while the broadening is around 100 meV (∼807 cm^–1^) at room temperature.^49^ Therefore, the Placzek approximation holds for our specific case. This is why we adopted it in this work to corroborate the experimental resonant Raman data. Indeed, as shown below, the experimental and calculated REPs for WS_2_ and WSSe are in reasonably good agreement. Nevertheless, the Placzek approximation misses key physics like intermediate states and multiple vibrational excitations. For resonant Raman, the third-order perturbation theory (Kramers–Heisenberg–Dirac (KHD) formula and Loudon’s theory) is more generalized and can directly take the intermediate states into account.^50–53^ In many works adopting the third-order perturbation theory in Raman scattering,^54,55^ the electron–photon and electron–phonon matrix elements along the electronic intermediate states are computed at the DFT level. Direct computation of the coupling matrices at the *GW* + BSE level to incorporate many-body effects is nontrivial to implement, except for a very recent work based on the time-dependent adiabatic *GW* theory using EPW and BERKELEYGW packages.^56^ On the other hand, the method based on calculations of the dielectric function can be implemented relatively more simply in DFT and *GW* + BSE packages for including the excitonic and electronic correlation effects. This may explain why the Placzek approximation is frequently used.

## Results and Discussion

### Photoluminescence and Raman Spectra of WS_2(1–*x*)_Se_2*x*_

To confirm that WS_2(1–*x*)_Se_2*x*_ intermediate Janus structures are stable and exhibit a typical linear bandgap decrease with *x*, we measured their PL spectra at 0 ≤ *x* ≤ 0.5. Figure 1b shows a set of PL spectra measured with 532 nm excitation for *x* = 0 (WS_2_), 0.14, 0.25, 0.38, and 0.5 (WSSe), which is used for REP investigation in this study. The center position of the PL bands decreases linearly with *x* from 2.020 eV (*x* = 0) to 1.838 eV (*x* = 0.5) with a slope of −0.37 eV/*x* demonstrating 182 meV bandgap tuning (Figure 1c), which is important for optoelectronic applications. Interestingly, the width of the bands goes through a maximum at *x* = 0.25, when half of the WS_2_ monolayer crystal is converted to Janus, followed by a decrease with *x* (Figure 1d). This can be related to the most disordered alloy crystal synthesized at this conversion fraction. It has been shown by atomic-resolution STEM in our previous work^8^ that, in this case, the intermediate Janus structure exhibits mainly vacancy–vacancy (V–V) and V–S defects without distortion of its structural integrity.

Figure 2 shows the evolution of the Raman spectra of WS_2(1–*x*)_Se_2*x*_ with *x*. Here, we focus mainly on the investigation of REPs of two phonon modes, *A*_1_ of WSSe and $A_{1}^{\prime }$ of WS_2_, which are highlighted in Figure 2. The *A*_1_( $A_{1}^{\prime })$ modes of WSSe and WS_2_ exhibit a shift depending on *x*, which has been investigated in detail in our previous study.^8^ The *A*_1_ WSSe mode frequency increases linearly with a slope of 49.7 cm^–1^/*x,* as can be seen from the inset in Figure 2, and the frequency of the $A_{1}^{\prime }$ mode of WS_2_ decreases only slightly with *x*.

**Figure 2:**
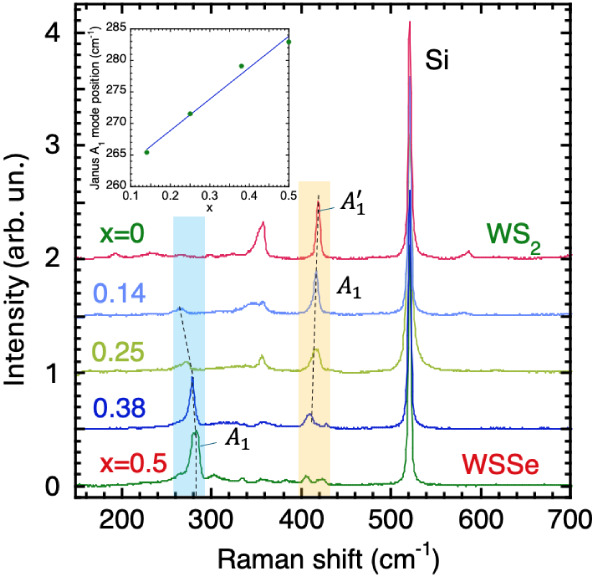
Evolution of Raman spectra of WS_2(1–*x*)_Se_2*x*_ with *x*. Raman spectra of the WS_2_ monolayer before conversion to Janus (*x* = 0), after conversion to WSSe (*x* = 0.5), and Janus intermediates (*x* = 0.14, 0.25, and 0.38) measured at 490 nm excitation. The shadowed regions highlight spectral shifts of the $A_{1}^{\prime }$ Raman mode of WS_2_ at 418 cm^–1^ and *A*_1_ mode of WSSe (282 cm^–1^) with the fraction, *x*. The dashed lines track the Raman peak positions at different *x*. All the spectra are normalized to the intensity of the Si line at 521 cm^–1^ and are offset vertically for clarity. The inset shows the *A*_1_ mode position versus *x* that increases linearly with a slope of 49.7 cm^–1^/*x*. The solid red circles are the experimental points, and the blue line is a linear fit.

### Raman Spectra at Different Excitation Wavelengths—REPs

First, we discuss measurements and calculations of Janus WSSe REPs, followed by REPs of fractional Janus monolayers, WS_2(1–*x*)_Se_2*x*_, with *x* = 0.14, 0.25, and 0.38. Figure 3a, b shows how the Raman spectra of Janus WSSe (*x* = 0.5) vary as the excitation wavelength is tuned from 460 to 575 nm. At 460 nm excitation, the Raman spectrum mainly consists of two pronounced first-order *A*_1_ modes at 282 cm^–1^ and 406 cm^–1^. As the excitation wavelength increases second-order Raman modes appear with the most intense 2LA­(M) mode at 305 cm^–1^ dominating the Raman spectrum (detailed assignment of the WSSe Raman modes is reported in refs. 7,8 . Figure 3c presents REPs of the two most intense Raman modes, *A*_1_ (282 cm^–1^) and 2LA­(M) (305 cm^–1^) that were derived from the Raman spectra in Figure 3a as the ratio of the integrated intensities of these modes to the integrated intensity of the 521 cm^–1^ Si Raman line. The REP of the *A*_1_ mode exhibits two pronounced peaks at 480 and 532 nm that are tentatively assigned to the excitons C (X_C_) and B (X_B_) in WSSe. Interestingly, the REP of the 2LA­(M) mode also shows two peaks that are slightly shifted, with the reverse intensity ratio; i.e., the peak corresponding to the X_B_ is the most intense feature in the REP of the 2LA­(M) mode. This difference in the REPs suggests that the exciton–phonon coupling is clearly phonon mode-dependent. In this study, we did not perform a fine scan of the excitation wavelength of the REP using the 2LA­(M) Raman mode since we mainly focused on the first-order modes. The reason for this is that theoretical interpretation of REPs of the second-order modes is more complex and cannot be conducted using our first-order Raman simulations approach. Because of the rough scan in this case, it is hard to make a definite conclusion about the actual shift of the 2LA­(M) mode REP.

**Figure 3:**
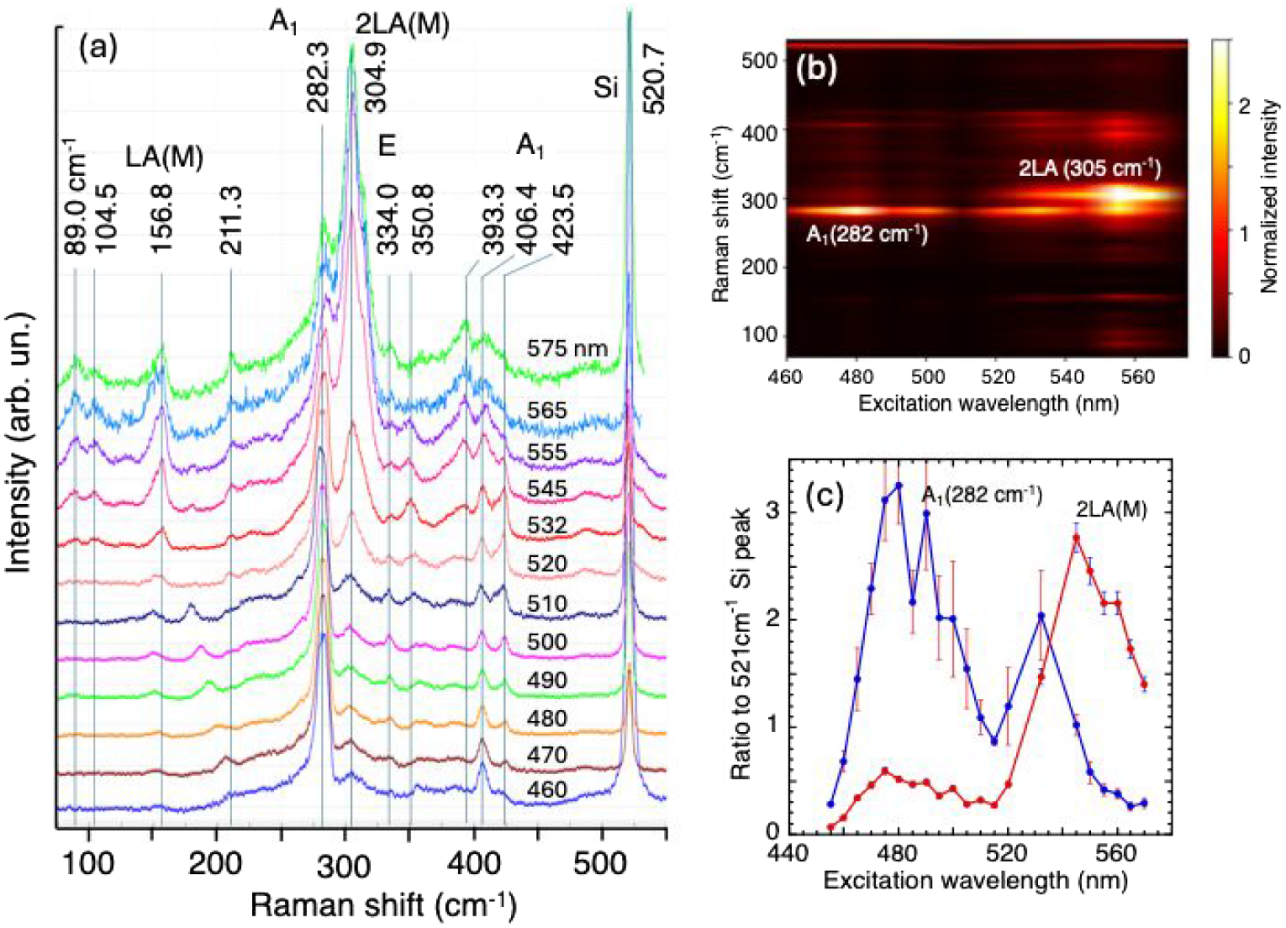
Evolution of Raman spectra of Janus WSSe with excitation wavelength: Raman excitation profiles (REPs). (a) A set of WSSe Raman spectra measured in the range of laser excitation wavelengths from 460 to 575 nm on a SiO_2_/Si substrate. The intensities are normalized to the 282 cm^–1^ Raman peak after the removal of the flat background. (b) Evolution of Raman peak intensities with excitation wavelength in the color map. (c) Raman excitation profiles of the *A*_1_ (282 cm^–1^) and 2LA­(M) (305 cm^–1^) modes of a WSSe monolayer crystal derived from the Raman spectra in (a) as the ratio of the integrated intensities of these modes to that of the 521 cm^–1^ Si Raman line.

### REP Corrections

However, the REP of a specific Raman mode, *m*, obtained as the ratio of its integrated intensity, *I*(*m*,*E*_*ph*_) (*E*_*ph*_ is the excitation photon energy), to that of the first-order Si (100) mode at 521 cm^–1^, *I*(521 cm^–1^ Si, *E*_*ph*_), needs to be corrected due to two effects. The first correction comes from the resonant character of the 521 cm^–1^ Si Raman mode itself^57–60^ (see note S2 in the SI). The second correction is related to the interference in a TMDC/SiO_2_/Si multilayer system, which is determined by the refractive indices of the layers and the corresponding phase factors involving the layer thicknesses through Fresnel’s equations, applied for our normal incidence irradiation geometry and backscattering detection of the Raman light (see note S3 in the SI). This approach has been widely used to predict enhancement factors of Raman scattering^61–63^ and photoluminescence (PL)^63^ as well as to correct REPs for the interference^21,23,26^ of both the incident excitation beam and the Raman scattered light from a TMDC layer and SiO_2_/Si. Note that calculations of the enhancement factors obviously require comparison of Raman (PL) signals from TMDC/SiO_2_/Si with those from the free-standing TMDC layers, which are commonly achieved by normalizing a predicted interference dependence to that calculated from a free-standing film.^62,63^ However, corrections of the REPs are simply based on the ratio of the Raman intensities from Si (521 cm^–1^) and a specific mode of a TMDC film modified by interference. Therefore, interference correction factors calculated using normalized intensities^26^ could lead to incorrect results.

Since the complex refractive indices of Janus WSSe monolayer crystals and their fractional intermediates are not known, we calculated these indices based on the fractional averaging of the known indices *ñ*_WS2_(*E*_*ph*_) and *ñ*_WS2_(*E*_*ph*_) (see note S3 in the SI). Figure 4 gives an example of the effect of the Si cross-section and both *σ*_*Si*_ and interference corrections on the REP of the $A_{1}^{\prime }$ mode of the WS_2_ monolayer at 418 cm^–1^, *I*(418 cm^–1^ WS_2_,*E*_*ph*_)/*I*(521 cm^–1^ Si,*E*_*ph*_). These corrections strongly affect the REP at the excitation photon energies *E_ph_* > 2.5 eV, where X_C_ is located.

**Figure 4:**
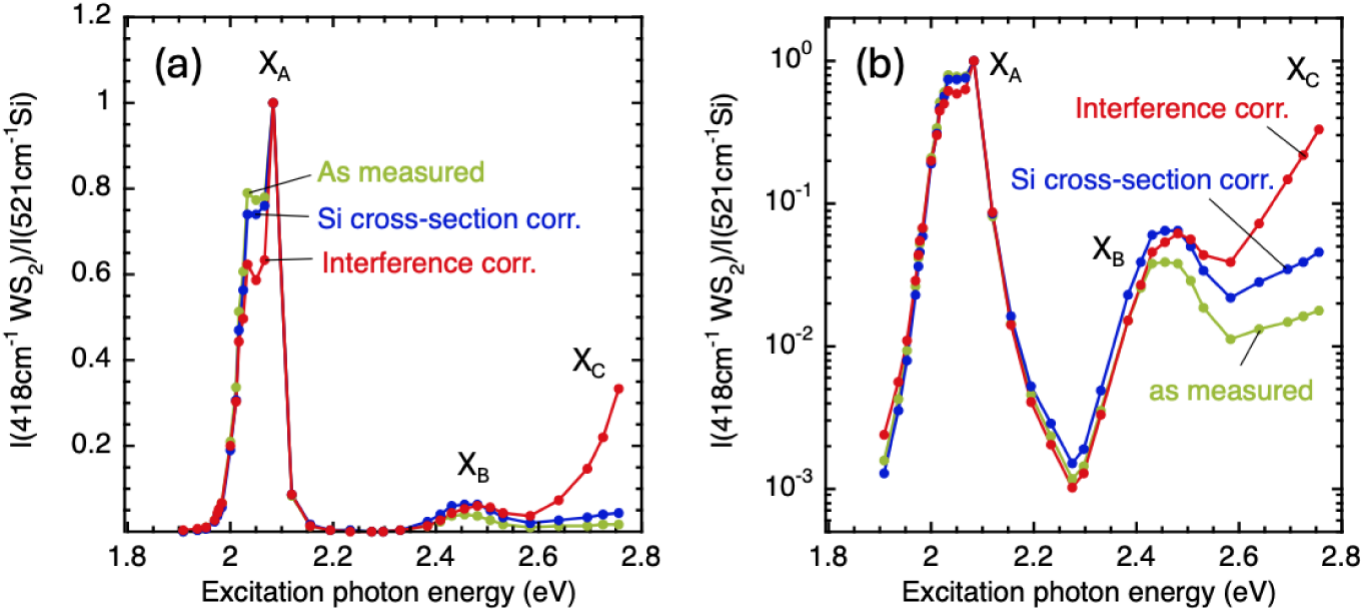
Illustration of the effects of substrate-related spectral corrections on REPs. (a) Linear and (b) semilogarithmic scales. The three curves correspond to the as measured ratio, *R*_0_ = *I*(418 cm^–1^ WS_2_,*E*_*ph*_)/*I*(521 cm^–1^Si,*E*_*ph*_) (green), Si cross-section (blue), and both *σ*_*Si*_ and interference (red) corrected REPs of the $A_{1}^{\prime }$ mode of the WS_2_ monolayer at 418 cm^–1^. All curves are normalized to the X_A_ peak.

The relatively large difference between the as-measured and corrected REPs in the spectral region around 2.05 eV is related to the very small intensities of both the 521 cm^–1^ Si and the 418 cm^–1^ $A_{1}^{\prime }$ WS_2_ monolayer Raman lines, due to strong absorption at the position of the X_A_ resonance, which increases the measurement error due to the small signal-to-noise ratio (see Figure S3 in the SI). The X_A_ peak shows two components separated by ∼40 meV (see also Figures 5a and 6a) and a tail at lower photon energies, which is consistent with exciton–trion (X_A_–X_A_^–^) contributions, the REPs of which have been studied in detail by McDonnell et al.^30^

**Figure 5:**
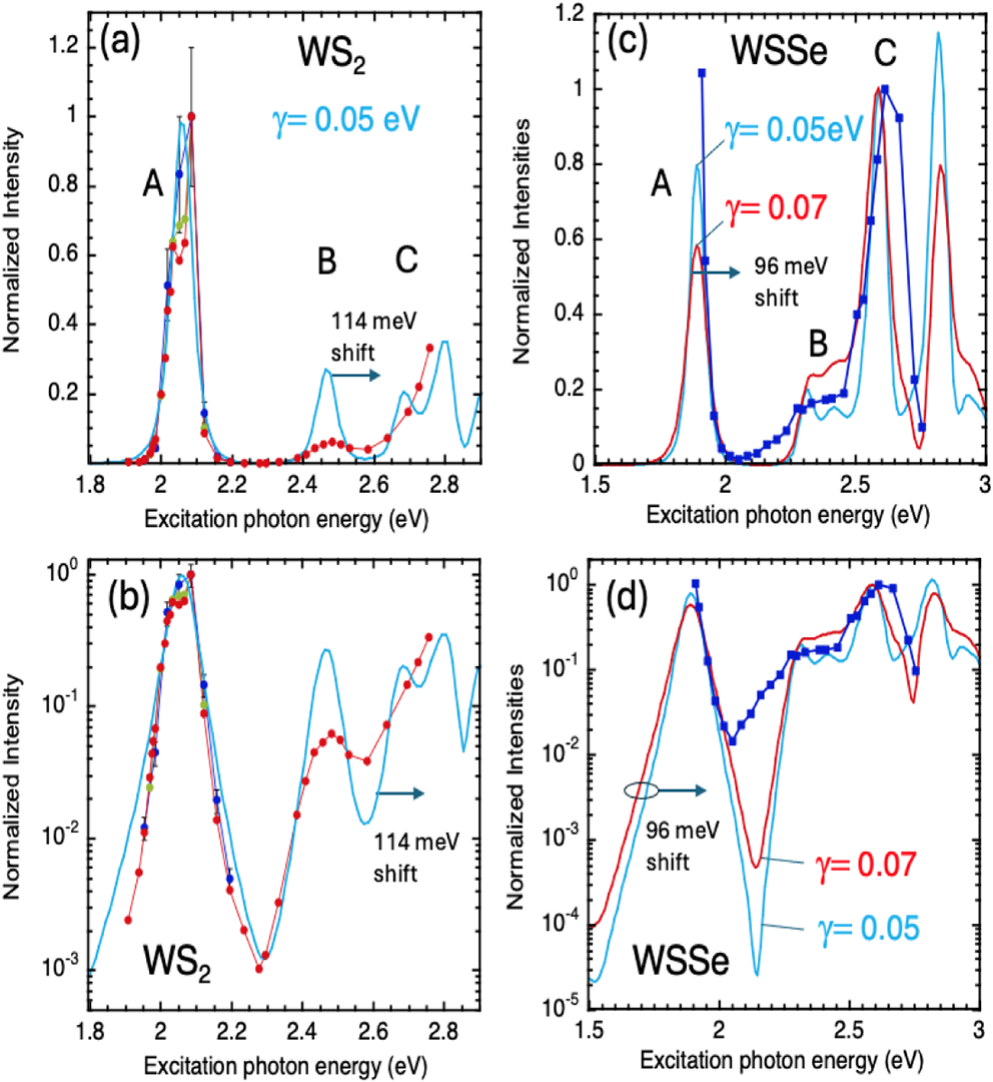
Comparison of experimental and calculated Raman excitation profiles (REPs) for WS_2_ and WSSe. (a), (b) REP of the $A_{1}^{\prime }$ (418 cm^–1^) mode of WS_2_ monolayer crystal plotted in linear (a) and semilogarithmic (b) scales. The solid circles correspond to measured and corrected REPs, and the light blue solid line presents calculated REPs with γ = 0.05 eV. Different circle colors (red, blue, and green) correspond to three different data sets. (c), (d) REPs of the *A*_1_ (282 cm^–1^) mode of WSSe monolayer crystal plotted in linear (c) and semilogarithmic (d) scales. The red and light blue curves correspond to calculations with γ = 0.07 and 0.05 eV, respectively. The solid blue squares correspond to experimental data. Note that the calculated REPs are shifted by 114 meV for WS_2_ and 96 meV for WSSe to higher energies to match the experimental points.

**Figure 6:**
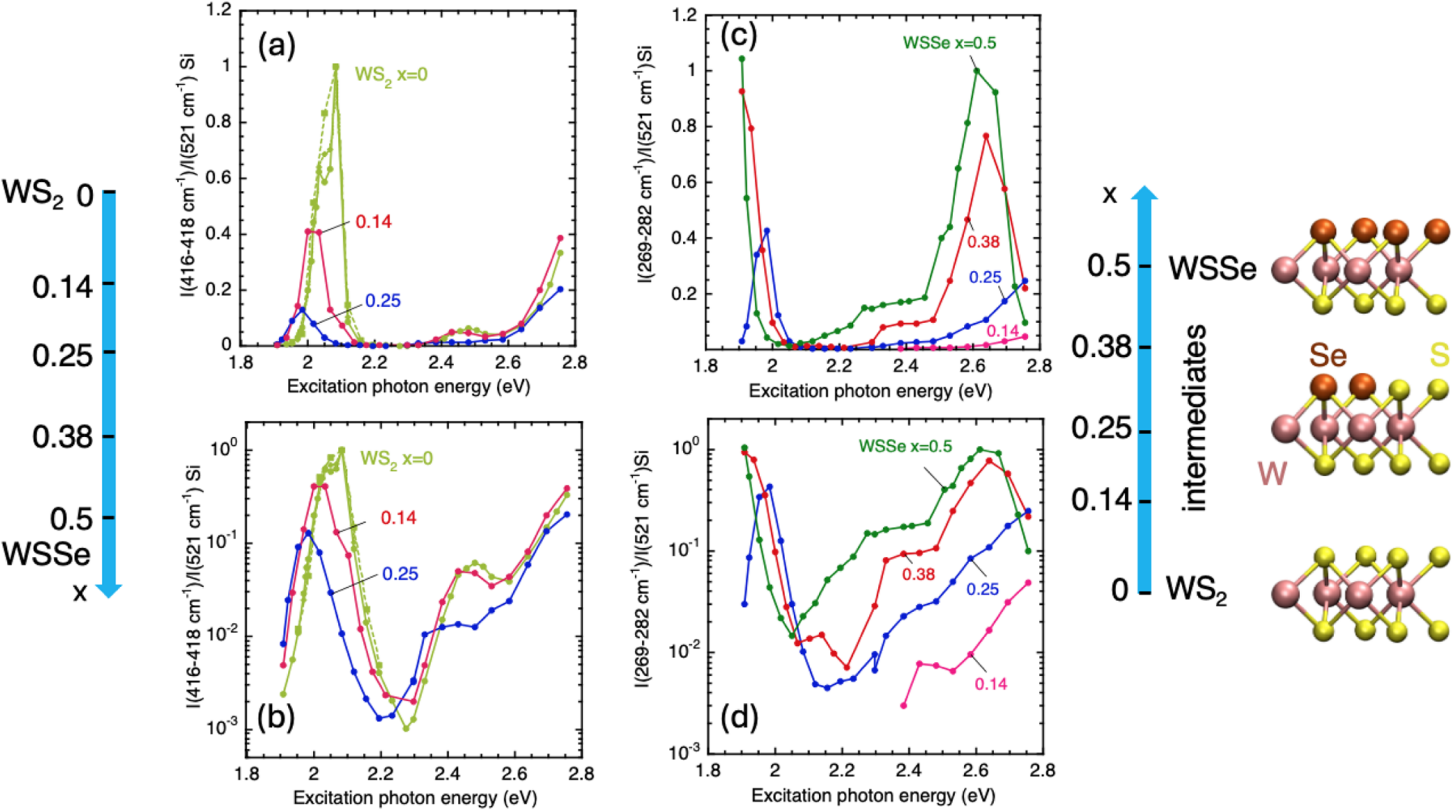
Raman excitation profiles of Janus intermediates. (a, b) Evolution of Raman excitation profiles of the $A_{1}^{\prime }$ (418 cm^–1^) mode of WS_2_ monolayer and (c, d) the *A*_1_ (282 cm^–1^) mode of WSSe by stepwise conversion from WS_2_ monolayer to WSSe by PLD. The fractional Janus monolayers, WS_2(1‑*x*)_Se_2*x*_, with *x* = 0.14, 0.25, and 0.38, are obtained using calibrated and arrested PLD of a Se target, as described in ref.^8^. A semilogarithmic scale is used in (b, d). The inset on the right shows side views of the Janus WSSe monolayer (*x* = 0.5), the partially converted monolayer (*x* = 0.25), and original WS_2_ structures.

### First-Principles Raman Simulations

To understand the origin of the measured REPs, we carried out first-principles Raman simulations of monolayer TMDs and the Janus structures, including WS_2_ and WSSe. The Raman intensity of any phonon mode is closely related to its Raman tensor according to the Placzek approximation (see Computational Details and note S1 in the SI).^35–38^ In the frozen-phonon limit, to obtain the Raman tensor, one basically needs to calculate the derivatives of the dynamic dielectric tensor with respect to the atomic displacements.^28,49^ To accurately calculate the dynamic dielectric tensor as a function of the electronic excitation energy, it is necessary to perform calculations at the many-body *GW +* BSE level.^45–48^ This should allow capturing the electron–electron and electron–hole interactions in the resonant Raman scattering of monolayer TMDs in order to quantitatively describe the electronic resonance and excitonic effects in the computed REPs. As a result, the calculated excitation energy can be almost directly compared to the experimental laser photon energy, with a difference of less than 0.2 eV. Note that calculations of the dynamic dielectric tensor need to be repeated for the perturbed system by different atomic displacements along ±*x*, ±*y,* and ±*z* directions using the *GW* + BSE framework, so the derivatives of the dynamic dielectric tensor can be obtained. *GW* + BSE calculations of an equilibrium system are already much more computationally expensive than DFT, and resonant Raman calculations at the *GW* + BSE level further increase the computational cost exponentially. The spin–orbit coupling (SOC) must be included to account for the valence band splitting to properly describe A and B excitons in TMDs^64–66^ rendering the calculations even more time-consuming.

### Experiment–Calculations Comparison

Figure 5 compares experimental and calculated REPs for WS_2_ (a, b) and WSSe (c, d). Note that the calculated REPs are shifted by 114 meV for WS_2_ and 96 meV for Janus WSSe to higher excitation photon energies. The WS_2_ $A_{1}^{\prime }$ mode REP is similar to that measured by Del Corro et al.,^28^ showing the strongest REP peak at 2.05 eV, which corresponds to the X_A_. The shifted theory curves (light blue in Figure 5a, b) predict well the X_A_ and X_B_ band positions in the REPs and their widths, with γ = 0.05 eV. Note that γ is a parameter in our calculations that sets a Lorentzian broadening in the dielectric function, which governs the width of the peaks in the REPs and is related to the lifetimes of the excitons. However, our calculations do not reproduce well the X_A_/X_B_ REP bands’ intensity ratio. A possible contributing factor for this is that the Raman simulations correspond to the condition of 0 K temperature, while experimental Raman measurements were performed at room temperature, where the spin–orbit coupling strength and electronic band structure are somewhat different. Due to the limited tunability range of our laser (1.91–2.76 eV), we could not measure the complete REP of the X_C_ and resolve its structure as predicted by the calculations.

Our calculations also well predict the main features of the REP of the *A*_1_ (282 cm^–1^) mode of Janus WSSe monolayer, mainly in the range of 2.0–2.8 eV, which by analogy with WS_2_ REPs, can be tentatively assigned to X_B_ and X_C_. However, in this case, the REPs (Figure 5c, d) are broader that require an increasing γ, which is likely due to the reduced crystallinity of the Janus WSSe monolayer compared to the WS_2_ monolayer. The PLD conversion from WS_2_ to WSSe unavoidably introduces defects, leading to broader Raman bands and REPs. In addition, the structural asymmetry of WSSe monolayers can also contribute to the broadening.^67^ Therefore, we used larger broadening parameters (γ) in our simulations to compare with the experimental REPs. Although larger broadening gives rise to a better match with the experimental data, the spectral resolution is reduced, so the structure in the calculated X_B_ WSSe REP is not completely resolved when γ = 0.07 eV, like in the experimental REP, where the X_B_ peak is not distinct (Figure 5c, d). However, the calculations conducted with a smaller γ (0.05 eV) better reveal the structure in the X_B_ WSSe REP. These results suggest that first-principles resonant Raman simulations can provide predictive energies and resolution for excitons that may be beyond the capability of experimental resonant Raman spectroscopy, so they can be a complementary tool to study resonant Raman behavior. The tunability range of our laser did not allow us to get the complete REP for the X_A_; nevertheless, based on the partially measured REP, we can conclude that all three X_A_, X_B_, X_C_ of Janus WSSe are strongly coupled to the *A*_1_ phonon, like the case for WS_2_. Such strong exciton–phonon coupling of the *A*_1_ phonon is also clearly predicted by our simulations.

To gain more insights on the exciton–phonon coupling, we computed optical oscillator strengths and exciton–phonon coupling strengths of monolayer WS_2_ and WSSe based on *GW* + BSE with SOC included. For first-order resonant Raman scattering, the Raman intensity scales according to $I_{\mathrm{Raman}}\propto {f_{S}}^{2}\cdot \gamma_{\mathrm{ex‐ph}}^{2}$ , where *f*_*S*_ is the oscillator strength of the exciton transition and *γ*_*ex‑ph*_ is the exciton–phonon coupling strength. Therefore, the exciton–phonon coupling strength $\gamma_{\mathrm{ex‐ph}}\propto \frac{\sqrt{I_{\mathrm{Raman}}}}{f_{S}}$ . With the calculated intensity of a Raman mode and oscillator strength at the energies of A, B, and C excitons, we can determine how *γ*_*ex‑p* *h*_ evolves from A to C exciton for this Raman mode, as shown in Table 1. For the WS_2_ $A_{1}^{\prime }$ mode, *γ*_*ex‑ph*_ decreases from A to B exciton by −10.6%, and then increases from A to C exciton by roughly 12.9%. For the WSSe *A*_1_ mode, on the other hand, *γ*_*ex‑ph*_ continues to grow from X_A_ to X_B_ to X_C_, where the total amount of increase is about 64.2% from A to C exciton. From WS_2_ to WSSe, *γ*_*ex‑ph*_ (X_A_) and *γ*_*ex‑ph*_ (X_B_) are both weakened, where *γ*_*ex‑ph*_ (X_A_) for the A exciton is noticeably reduced by −27.3%. On the contrary, *γ*_*ex‑ph*_ (X_C_) for the C exciton is increased by 5.7% from WS_2_ to WSSe. These trends in exciton–phonon coupling strengths, coupled with the changes in oscillator strengths, can shed light on the REPs observed in Figure 5. For example, for the WS_2_ $A_{1}^{\prime }$ mode, *γ*_*ex‑ph*_ does not change much from A to B exciton, while the oscillator strength is reduced almost by half, and thus the Raman intensity is reduced by nearly 75% from A to B exciton (recall $I_{\mathrm{Raman}}\propto {f_{S}}^{2}\cdot \gamma_{\mathrm{ex‐ph}}^{2}$ ). From A to C exciton, the mild increase of *γ*_*ex‑ph*_ by roughly 12.9% does not compensate enough for the significant reduction of the oscillator strength by 46.9%, and hence the intensity is notably weakened. As for the WSSe *A*_1_ mode, one similarity is that *γ*_*ex‑ph*_ does not increase much from A to B exciton, while the oscillator strength is reduced by more than half, and thus the Raman intensity is reduced by 75% from A to B exciton. However, the trend from A to C exciton is very different for the WSSe *A*_1_ mode. Because *γ*_*ex‑ph*_ is significantly increased by 64.2% from A to C exciton, while the oscillator strength is only reduced by 31.8%, the intensity is now enhanced from A to C exciton for the WSSe *A*_1_ mode, the opposite of the trend of the WS_2_ $A_{1}^{\prime }$ mode. Our results explain why *I*(X_A_) > *I*(X_C_) for WS_2_ $A_{1}^{\prime }$ mode, while they are comparable for the WSSe *A*_1_ mode in the experimental REPs shown in Figure 5. In short, the Se substitution affects both exciton–phonon coupling strengths and oscillator strengths, resulting in complicated changes in REPs during the Janus conversion process.

**Table 1:** Calculated Exciton–Phonon Coupling Strengths *γ*_*ex‑ph*_ of Monolayer WS_2_ ( $A_{1}^{\prime }$ mode) and WSSe (*A*_1_ Mode) versus Different Excitons.^a^

**WS**_**2**_				**WSSe**			
		$A_{1}^{\prime }$ mode				A_1_ mode	
Exciton (energy)	oscillator strength	*γ*_*ex‑ph*_	*I*_Raman_	Exciton (energy)	oscillator strength	*γ*_*ex‑p*h_	*I*_Raman_
X_A_ (1.94 eV)	23128.5	**0.00388**	8063.4	X_A_ (1.79 eV)	24192.6	**0.00282**	4671.3
X_B_ (2.35 eV)	13588.9	**0.00347**	2233.6	X_B_ (2.22 eV)	10700.1	**0.00319**	1172.2
X_C_ (2.68 eV)	12273.3	**0.00438**	2890.1	X_C_ (2.49 eV)	16507.2	**0.00463**	5862.3

a Calculated oscillator strengths *f*_*S*_ of different excitons are also shown The formula used is $\gamma_{\mathrm{ex‐ph}}\propto \frac{\sqrt{I_{\mathrm{Raman}}}}{f_{S}}$ . Since we cannot directly compute the exciton−phonon coupling matrix elements, we focus on how calculated exciton−phonon coupling strengths evolve with different excitons and phonon modes, instead of their absolute values. Note that the calculated exciton energies are slightly lower than experimental values measured in REPs, as shown in Figure 5

### REP Evolution with the Conversion Factor, *x*

Figure 6 shows how the REPs of the $A_{1}^{\prime }$ (418 cm^–1^) mode of WS_2_ monolayer and the *A*_1_ (282 cm^–1^) mode of WSSe evolve during conversion from WS_2_ to WSSe by PLD. As can be seen from Figure 6a, the X_A_ REP intensity of the $A_{1}^{\prime }$ (418 cm^–1^) mode of WS_2_ gradually decreases, and the center frequency shifts to lower photon energies as the fraction, *x*, changes from *x* = 0 to *x* = 0.25. The REP of the X_B_ behaves similarly but becomes less pronounced, converting to a structureless broad band overlapping with the REP of the X_C_. In this case, we were unable to measure the REP at *x* = 0.38 due to the low intensity of the corresponding Raman band. A new Raman line at ∼265 cm^–1^ appears at a small conversion fraction of 0.14 and gradually shifts to ∼283 cm^–1^ as the fraction increases to 0.5, corresponding to the Janus WSSe monolayer crystal (Figure 2).

This Raman mode has the vibration mostly concentrated on the substitutional Se atoms and has been predicted by our prior calculations and measured experimentally.^8^ Its Raman peak position is almost linearly dependent on the fraction *x* (i.e., the Se content) and thus can be used to track the structural evolution during PLD conversion. Figure 6c, d shows its REPs measured at different conversion fractions: *x* = 0.14, 0.25, 0.38, and 0.5 (WSSe). Interestingly, even at the conversion fraction of 0.25, when only half of the S atoms in the WS_2_ top layer are replaced with Se, a clear peak corresponding to the REP of the X_A_ appears, while only a broad REP feature emerges at the spectral positions of the X_B_ and X_C_. However, at *x* = 0.38, three REP bands appear, resembling those for the X_A_, X_B_, and X_C_ of Janus WSSe, but slightly shifted in frequency. At this conversion fraction, the REP of the X_C_ emerges as a strong band comparable to that of X_A_. For Janus WSSe (*x* = 0.5), its three exciton positions are downshifted compared to those at *x* = 0.38, as shown in Figure 6c, d. In short, the energy downshift of the X_A_, X_B_, and X_C_ excitons with the increasing fraction *x* from 0 to 0.5 is consistent with the trends reported in the literature^28^ that the A, B, and C exciton energies are lowered from monolayer WS_2_ to WSe_2_. Such a downshift has also been previously established for A-excitons during the conversion of MoS_2_ to MoSSe through Janus alloys, MoS_2(1–*x*)_Se_2*x*_, based on photoluminescence measurements.^14^ Our REP measurements of the intermediate Janus structures reveal the gradual evolution of such energy shifts for all three excitons.

## Conclusions

In this study, we target important optoelectronic applications of Janus 2D nanomaterials, such as solar cells and photodetectors.^9^ Both these applications require a detailed understanding of the excitonic structure of these materials, which can be effectively investigated by REPs. However, experimental REP studies are limited by the tuning range of existing CW lasers needed for resonance Raman spectroscopy. Therefore, developing a reliable theory that can predict REPs for any range of photon energies will be beneficial for these applications.

Tunable resonant Raman excitation is employed to investigate Raman excitation profiles of Janus WSSe monolayers and their intermediate alloy structures synthesized by *in situ* Raman-controlled PLD. The resonant REPs of the $A_{1}^{\prime }$ WS_2_ and *A*_1_ WSSe Raman modes reveal excitonic transitions, exciton–phonon coupling, and the transformation of the excitonic bands during the conversion of WS_2_ monolayers to Janus WSSe structures. The intermediate Janus structures, WS_2(1‑*x*)_Se_2*x*_ with *x* = 0.14, 0.25, and 0.38, are synthesized using laser ablation of a Se target by controlling the number of laser pulses with *in situ* Raman scattering. These intermediate Janus alloy structures are stable and exhibit pronounced resonant REPs, which show that all three excitonic bands transform from WS_2_ to WSSe through a gradual shift of the A, B, and C excitonic bands to lower frequencies.

To understand the measured REPs, we conducted first-principles resonant Raman simulations, which are in good agreement with the experimental results, corroborating that the A-symmetry Raman modes in WS_2_ and WSSe are strongly coupled to the A, B, and C excitons. The good agreement of the calculations with the experimental results indicates that first-principles resonant Raman simulations can provide predictive energies and spectral resolution for REPs that may be beyond the capability of experimental resonant Raman spectroscopy due to the limited tunability range of existing lasers needed for Raman excitation. Therefore, the validation of our first-principles resonant Raman simulations shows that such simulations can serve as a complementary predictive tool for studying resonant Raman behavior. This study provides a nondestructive, quantitative pathway for deterministic bandgap and electronic-state engineering in Janus/alloyed TMDC monolayers.

## Supplementary Material



## Notes

Note S1, Computational details for phonon and Raman simulations; Note S2, Correction for resonant Si Raman scattering; Note S3, Interference correction factor; Figures S1–S3 (PDF)

## #

Department of Materials Science and Engineering, National Yang Ming Chiao Tung University, Hsinchu City, Taiwan, 300393

## References

[1] Butler S. Z., Hollen S. M., Cao L., Cui Y., Gupta J. A., Gutiérrez H. R., Heinz T. F., Hong S. S., Huang J., Ismach A. F., Johnston-Halperin E., Kuno M., Plashnitsa V. V., Robinson R. D., Ruoff R. S., Salahuddin S., Shan J., Shi L., Spencer M. G., Terrones M., Windl W., Goldberger J. E. (2013). Progress, Challenges, and Opportunities
in Two-Dimensional Materials Beyond Graphene. ACS Nano.

[2] Bhimanapati G. R., Lin Z., Meunier V., Jung Y., Cha J., Das S., Xiao D., Son Y., Strano M. S., Cooper V. R., Liang L., Louie S. G., Ringe E., Zhou W., Kim S. S., Naik R. R., Sumpter B. G., Terrones H., Xia F., Wang Y., Zhu J., Akinwande D., Alem N., Schuller J. A., Schaak R. E., Terrones M., Robinson J. A. (2015). Recent Advances in Two-Dimensional Materials beyond
Graphene. ACS Nano.

[3] Susarla S., Kutana A., Hachtel J. A., Kochat V., Apte A., Vajtai R., Idrobo J. C., Yakobson B. I., Tiwary C. S., Ajayan P. M. (2017). Quaternary 2D Transition
Metal Dichalcogenides (TMDs)
with Tunable Bandgap. Adv. Mater.

[4] Riis-Jensen A. C., Deilmann T., Olsen T., Thygesen K. S. (2019). Classifying
the
Electronic and Optical Properties of Janus Monolayers. ACS Nano.

[5] Manzeli S., Ovchinnikov D., Pasquier D., Yazyev O. V., Kis A. (2017). 2D transition
metal dichalcogenides. Nat. Rev. Mater..

[6] Dong L., Lou J., Shenoy V. B. (2017). Large In-Plane
and Vertical Piezoelectricity in Janus
Transition Metal Dichalchogenides. ACS Nano.

[7] Lin Y. C., Liu C. Z., Yu Y. L., Zarkadoula E., Yoon M., Puretzky A. A., Liang L. B., Kong X. R., Gu Y. Y., Strasser A., Meyer H., Lorenz M., Chisholm M. F., Ivanov I. N., Rouleau C. M., Duscher G., Xiao K., Geohegan D. B. (2020). Low Energy
Implantation into Transition-Metal
Dichalcogenide Monolayers to Form Janus Structures. ACS Nano.

[8] Harris S. B., Lin Y. C., Puretzky A. A., Liang L. B., Dyck O., Berlijn T., Eres G., Rouleau C. M., Xiao K., Geohegan D. B. (2023). Real-Time Diagnostics
of 2D Crystal Transformations
by Pulsed Laser Deposition: Controlled Synthesis of Janus WSSe Monolayers
and Alloys. ACS Nano.

[9] Ahmad W., Wang Y., Kazmi J., Younis U., Mubarak N. M., Aleithan S. H., Channa A. I., Lei W., Wang Z. M. (2025). Janus 2D
Transition Metal Dichalcogenides: Research Progress, Optical Mechanism
and Future Prospects for Optoelectronic Devices. Laser Photonics Rev..

[10] Xie L. M. (2015). Two-dimensional
transition metal dichalcogenide alloys: Preparation, characterization
and applications. Nanoscale.

[11] Chaves A., Azadani J. G., Alsalman H., da Costa D. R., Frisenda R., Chaves A. J., Song S. H., Kim Y. D., He D. W., Zhou J. (2020). Bandgap
engineering of two-dimensional semiconductor
materials. Npj 2D Mater. Appl..

[12] Bouaziz M., Schue L., Andriambelaza N. F., Alyabyeva N., Girard J. C., Dudin P., Cadiz F., Avila J., Dappe Y., Gonzalez C. (2025). Tunable
electronic band
structure in WS_2(1‑x)_Se_2x_ van der Waals
alloys. Phys. Rev. B.

[13] Ernandes C., Khalil L., Almabrouk H., Pierucci D., Zheng B. Y., Avila J., Dudin P., Chaste J., Oehler F., Pala M. (2021). Indirect to direct band gap crossover in two-dimensional
WSSe alloys. Npj 2D Mater. Appl..

[14] Schmeink J., Osterfeld J., Kharsah O., Sleziona S., Schleberger M. (2024). Unraveling
the influence of defects in Janus MoSSe and Janus alloys MoS_2(1‑x)_Se_2x_. Npj 2D Mater. Appl..

[15] Zhang X., Tan Q. H., Wu J. B., Shi W., Tan P. H. (2016). Review
on the Raman spectroscopy of different types of layered materials. Nanoscale.

[16] Zhang X., Qiao X. F., Shi W., Wu J. B., Jiang D. S., Tan P. H. (2015). Phonon and Raman
scattering of two-dimensional transition
metal dichalcogenides from monolayer, multilayer to bulk material. Chem. Soc. Rev..

[17] Saito R., Tatsumi Y., Huang S., Ling X., Dresselhaus M. S. (2016). Raman spectroscopy
of transition metal dichalcogenides. J. Phys-Condens
Mater..

[18] Pimenta M. A., Resende G. C., Ribeiro H. B., Carvalho B. R. (2021). Polarized Raman
spectroscopy in low-symmetry 2D materials: Angle-resolved experiments
and complex number tensor elements. Phys. Chem.
Chem. Phys..

[19] Kowalski J., Liang L. (2026). Raman Digital Twin of Monolayer Janus Transition Metal Dichalcogenides. ACS Appl. Mater. Interfaces.

[20] Scheuschner N., Ochedowski O., Schleberger M., Maultzsch J. (2012). Resonant Raman
profiles and μ-photoluminescence of atomically thin layers of
molybdenum disulfide. Phys. Status Solidi B.

[21] Carvalho B. R., Malard L. M., Alves J. M., Fantini C., Pimenta M. A. (2015). Symmetry-Dependent
Exciton-Phonon Coupling in 2D and Bulk MoS_2_ Observed by
Resonance Raman Scattering. Phys. Rev. Lett..

[22] Carvalho B. R., Malard L. M., Alves J. M., Fantini C., Pimenta M. A. (2016). Symmetry-Dependent
Exciton-Phonon Coupling in 2D and Bulk MoS_2_ Observed by
Resonance Raman Scattering (vol 114, 136403, 2015). Phys. Rev. Lett..

[23] Soubelet P., Bruchhausen A. E., Fainstein A., Nogajewski K., Faugeras C. (2016). Resonance effects in
the Raman scattering of monolayer
and few-layer MoSe_2_. Phys. Rev. B.

[24] Puretzky A. A., Liang L. B., Li X. F., Xiao K., Wang K., Mahjouri-Samani M., Basile L., Idrobo J. C., Sumpter B. G., Meunier V., Geohegan D. B. (2015). Low-Frequency Raman Fingerprints
of Two-Dimensional Metal Dichalcogenide Layer Stacking Configurations. ACS Nano.

[25] Kim K., Lee J. U., Nam D., Cheong H. (2016). Davydov Splitting and
Excitonic Resonance Effects in Raman Spectra of Few-Layer MoSe_2_. ACS Nano.

[26] McCreary A., Simpson J. R., Wang Y. X., Rhodes D., Fujisawa K., Balicas L., Dubey M., Crespi V. H., Terrones M., Walker A. R. H. (2017). Intricate Resonant
Raman Response in Anisotropic ReS_2_. Nano Lett..

[27] Del
Corro E., Terrones H., Elias A., Fantini C., Feng S. M., Nguyen M. A., Mallouk T. E., Terrones M., Pimenta M. A. (2014). Excited Excitonic States in 1L, 2L, 3L, and Bulk WSe_2_ Observed by Resonant Raman Spectroscopy. ACS Nano.

[28] Del
Corro E., Botello-Méndez A., Gillet Y., Elias A. L., Terrones H., Feng S., Fantini C., Rhodes D., Pradhan N., Balicas L., Gonze X., Charlier J. C., Terrones M., Pimenta M. A. (2016). Atypical Exciton-Phonon
Interactions in WS_2_ and WSe_2_ Monolayers Revealed
by Resonance Raman Spectroscopy. Nano Lett..

[29] Molas M. R., Nogajewski K., Potemski M., Babinski A. (2017). Raman scattering excitation
spectroscopy of monolayer WS_2_. Sci.
Rep-Uk.

[30] McDonnell L. P., Huang C. C., Cui Q. S., Hewak D. W., Smith D. C. (2018). Probing
Excitons, Trions, and Dark Excitons in Monolayer WS_2_ Using
Resonance Raman Spectroscopy. Nano Lett..

[31] Kresse G., Furthmuller J. (1996). Efficiency of ab-initio total energy calculations for
metals and semiconductors using a plane-wave basis set. Comput. Mater. Sci..

[32] Togo A., Oba F., Tanaka I. (2008). First-principles
calculations of the ferroelastic transition
between rutile-type and CaCl_2_-type SiO_2_ at high
pressures. Phys. Rev. B.

[33] Placzek, G. Rayleigh Scattering and Raman Effect. In Handbuch der Radiologie, Marx, E. , Eds.;Akademische Verlagsgesellschaft: Leipzig, Germany, 1934; Vol. 6, pp. 205–374.

[34] Born, M. ; Huang, K. Dynamical Theory of Crystal Lattices; Oxford University Press: Oxford, U.K, 1954.

[35] Umari P., Pasquarello A., Dal Corso A. (2001). Raman scattering intensities in α-quartz:
A first-principles investigation - art. no. 094305. Phys. Rev. B.

[36] Kong X. R., Ganesh P., Liang L. B. (2024). First-principles
study of the magneto-Raman
effect in van der Waals layered magnets. Npj
2D Mater. Appl..

[37] Ceriotti M., Pietrucci F., Bernasconi M. (2006). Ab initio study of the vibrational
properties of crystalline TeO_2_: The α, β, and
γ phases. Phys. Rev. B.

[38] Liang L. B., Meunier V. (2014). First-principles Raman spectra of
MoS_2_,
WS_2_ and their heterostructures. Nanoscale.

[39] Ambrosch-Draxl C., Auer H., Kouba R., Sherman E. Y., Knoll P., Mayer M. (2002). Raman scattering
in YBa_2_Cu_3_O_7_: A
comprehensive theoretical study in comparison with experiments. Phys. Rev. B.

[40] Gillet Y., Giantomassi M., Gonze X. (2013). First-principles study of excitonic
effects in Raman intensities. Phys. Rev. B.

[41] Gillet Y., Gonze M., Giantomassi X., Draxl C., Gonze X. (2017). *Ab
Initio* Approach to Second-Order Resonant Raman Scattering
Including Exciton-Phonon Interaction. Sci. Rep..

[42] Niu L., Zhu J. Q., Gao W., Liu A. P., Han X., Du S. Y. (2008). First-principles
calculation of vibrational Raman spectra of tetrahedral
amorphous carbon. Phys. B.

[43] Wang Y., Carvalho B. R., Crespi V. H. (2018). Strong exciton regulation of Raman
scattering in monolayer MoS_2_. Phys.
Rev. B.

[44] Kammerlander T., Del Sole R., Marini A., Pulci O. (2019). Theory of resonant
Raman scattering: Towards a comprehensive ab initio description. Phys. Rev. B.

[45] Hedin L. (1965). New Method
for Calculating the One-Particle Green’s Function with Application
to the Electron-Gas Problem. Phys. Rev..

[46] Hybertsen M. S., Louie S. G. (1986). Electron Correlation in Semiconductors and Insulators:
Band Gaps and Quasiparticle Energies. Phys.
Rev. B.

[47] Strinati G. (1984). Effects of
Electron-Hole Interaction on the Inelastic Light Scattering in Semiconductors. Phys. Rev. B.

[48] Rohlfing M., Louie S. G. (2000). Electron-Hole Excitations
and Optical Spectra from
First Principles. Phys. Rev. B.

[49] Miranda H. P. C., Reichardt S., Froehlicher G., Molina-Sánchez A., Berciaud S., Wirtz L. (2017). Quantum Interference Effects in Resonant
Raman Spectroscopy of Single- and Triple-Layer MoTe_2_ from
First-Principles. Nano Lett..

[50] Kramers H. A., Heisenberg W. (1925). On the Scattering
of Radiation by Atoms. Z.Phys..

[51] Dirac P. A. M. (1927). The
Quantum Theory of the Emission and Absorption of Radiation. Proc. R. Soc. London A.

[52] Loudon R. (1963). The Raman
Effect in Crystals. Adv. Phys..

[53] Loudon, R. The Quantum Theory of Light; 3rd ed.; Oxford University Press: Oxford, U.K, 2000.

[54] Walter M., Moseler M. (2020). Ab Initio Wavelength-Dependent
Raman Spectra: Placzek
Approximation and Beyond. J. Chem. Theory Comput..

[55] Hung N. T., Huang J. Q., Tatsumi Y., Yang T., Saito R. (2024). QERAMAN: An
open-source program for calculating resonance Raman spectra based
on QUANTUM ESPRESSO. Comput. Phys. Commun..

[56] Chan Y. -H., Li Z. L., Louie S. G. (2025). Excitonic effects
on infrared vibrational
and Raman spectroscopy from first principles. Phys. Rev. B.

[57] Grimsditch M., Cardona M., Calleja J. M., Meseguer F. (1981). Resonance in the Raman-Scattering
of CaF_2_, SrF_2_, BaF_2_ and Diamond. J. Raman Spectrosc..

[58] Klar P., Lidorikis E., Eckmann A., Verzhbitskiy I. A., Ferrari A. C., Casiraghi C. (2013). Raman scattering efficiency of graphene. Phys. Rev. B.

[59] Renucci J. B., Tyte R. N., Cardona M. (1975). Resonant Raman-Scattering
in Silicon. Phys. Rev. B.

[60] Lee J. U., Park J., Son Y. W., Cheong H. (2015). Anomalous excitonic
resonance Raman effects in few-layered MoS_2_. Nanoscale.

[61] Wang Y. Y., Ni Z. H., Shen Z. X., Wang H. M., Wu Y. H. (2008). Interference
enhancement of Raman signal of graphene. Appl.
Phys. Lett..

[62] Yoon D., Moon H., Son Y. W., Choi J. S., Park B. H., Cha Y. H., Kim Y. D., Cheong H. (2009). Interference effect
on Raman spectrum of graphene on SiO_2_/Si. Phys. Rev. B.

[63] Zhang H., Wan Y., Ma Y. G., Wang W., Wang Y. L., Dai L. (2015). Interference
effect on optical signals of monolayer MoS_2_. Appl. Phys. Lett..

[64] Qiu D. Y., da Jornada F. H., Louie S. G. (2013). Optical Spectrum of MoS_2_: Many-Body Effects
and Diversity of Exciton States. Phys. Rev.
Lett..

[65] Komsa H. -P., Krasheninnikov A. V. (2012). Effects
of confinement and environment on the electronic
structure and exciton binding energy of MoS_2_ from first
principles. Phys. Rev. B.

[66] Shi H., Pan H., Zhang Y. -W., Yakobson B. I. (2013). Quasiparticle band
structures and
optical properties of strained monolayer MoS_2_ and WS_2_. Phys. Rev. B.

[67] Erkiliç U., Wang S. N., Sekine Y., Taniguchi T., Watanabe K., Taniyasu Y. (2025). Exciton Dynamics in Janus WSSe Driven
by Structural Asymmetry. Nano Lett..

