# Supplementary material for: Synthetic Tuning of Exciton–Phonon Coupling in Janus WS2(1‑x)Se2x Monolayers Revealed by Resonant Raman Excitation Spectroscopy for Optoelectronic Applications
Source: ACS Appl Nano Mater. 2026 Jun 17;9(25):11750–60. doi: 10.1021/acsanm.6c01104 (PMC13316852; doi:10.1021/acsanm.6c01104)
Supplement: Supplementary file 1 [file an6c01104_si_001.pdf]

SUPPORTING INFORMATION

Synthetic Tuning of Exciton-Phonon Coupling in Janus $WS_{2(1-x)}Se_{2x}$ Monolayers Revealed by Resonant Raman Excitation Spectroscopy for Optoelectronic Applications

Alexander A. Puretzky*[1], Liangbo Liang*[1], Sumner B. Harris[1], Yu-Chuan Lin[2#],

David B. Geohegan[3]

1. *Center for Nanophase Materials Sciences, Oak Ridge National Laboratory, Oak Ridge, Tennessee 37831, United States*
2. *Department of Materials Science and Engineering, The Pennsylvania State University, University Park, Pennsylvania 16802, United State*
3. *Department of Materials Science and Engineering, University of Tennessee, Knoxville, Tennessee 37996, United States*

[#] Current address: *Department of Materials Science and Engineering, National Yang Ming Chiao Tung University, Hsinchu City, Taiwan, 300393*

[*]Corresponding authors: puretzkya@ornl.gov; liangl1@ornl.gov

Note S1. Computational Details for Phonon and Raman Simulations

Plane-wave density functional theory (DFT) calculations were performed using the VASP package (version 5.4.4) equipped with the projector-augmented-wave (PAW) method for electron-ion interactions. [1] The exchange-correlation functional within local density approximation (LDA) was adopted to calculate phonon frequencies and vibrational patterns. For monolayer TMDs and the Janus systems, including WS_2 and WSSe, they were simulated by a periodic slab geometry with a vacuum separation distance of 21 Å in the out-of-plane direction (*z* direction) to avoid spurious interactions with periodic neighbor unit cells. Both the atomic positions and in-plane lattice constants were optimized until the residual forces were below 0.001 eV/Å. The energy cutoff was 400 eV and the k-point sampling was 24×24×1. Then the dynamic matrix was calculated in the finite difference scheme implemented in the Phonopy software. [2] Hellmann-Feynman forces in the 4×4×1 supercell were computed by VASP for both positive and negative atomic displacements (Δ = 0.03 Å) and then used in Phonopy to construct the dynamic matrix, whose diagonalization provides phonon frequencies and eigenvectors.

Raman intensities were calculated within the Placzek approximation using an open-source Python package "SpectroPy" developed by us (https://github.com/TheorySpectroPy/SpectroPy). For the j-th phonon mode, Raman intensity is $I \propto \frac{(n_j+1)}{\omega_j}|\boldsymbol{e}_\mathrm{i}\cdot\tilde{R}\cdot\boldsymbol{e}_\mathrm{s}^\mathrm{T}|^2$, where $\boldsymbol{e}_\mathrm{i}$ and $\boldsymbol{e}_\mathrm{s}$ are the electric polarization vectors of the incident and scattered light respectively, and $\tilde{R}$ is the Raman tensor of the phonon mode. ω_j is the frequency of the j-th phonon mode, and $n_j = (e^{\hbar\omega_j/k_BT} - 1)^{-1}$ is its Boltzmann distribution function at the given temperature $T = 300$ K. The matrix element of the (3×3) Raman tensor $\tilde{R}$ of the j-th phonon mode is [3-6]

$$\tilde{R}_{\alpha\beta}(j) = V_0\sum_{\mu=1}^{N}\sum_{l=x}^{z}\frac{\partial\chi_{\alpha\beta}}{\partial r_l(\mu)}\frac{e_l^j(\mu)}{\sqrt{M_\mu}}, \qquad \text{(S1)}$$

where $\chi_{\alpha\beta} = (\varepsilon_{\alpha\beta} - \delta_{\alpha\beta})/4\pi$ is the electric polarizability tensor related to the dielectric tensor $\varepsilon_{\alpha\beta}$, $r_l(\mu)$ is the position of the μ-th atom along the direction l, $\frac{\partial\chi_{\alpha\beta}}{\partial r_l(\mu)}$ is the derivative of the polarizability tensor (essentially the dielectric tensor) over the atomic displacement, $e_l^j(\mu)$

corresponds to the displacement of the μ-th atom along the direction l in the j-th phonon mode (i.e., the eigenvector of the dynamic matrix), M_μ is the mass of the μ-th atom, and V_0 is the unit cell volume. Basically, one needs to calculate the derivatives of the dielectric tensors with respect to the atomic displacements for obtaining the Raman tensors. To capture the dependence of Raman intensities on the laser excitation energy, we need to compute the dynamic dielectric tensors $\varepsilon_{\alpha\beta}$ as a function of the laser excitation energy (i.e., the dielectric function). Such a task can be realized at different approximation levels, using conventional DFT functionals like the LDA or the generalized gradient approximation (GGA) [7], or hybrid functionals [7], or the many-body perturbation theory [8, 9]. LDA and GGA functionals often underestimate the electronic band gap and electronic excitation energy. For quantitative comparison with experimental Raman data, it is necessary to calculate the dynamic dielectric tensors at the many-body level. In specific, the many-body perturbation theory within the *GW* approximation (*G* is the Green's function; *W* is the Coulomb interaction) is needed to obtain accurate electronic eigenvalues including unoccupied states, and solving the Bethe-Salpeter equation (BSE) is required to describe optical excitations and excitonic effects. This would allow us to capture many-body electron-electron and electron-hole interactions in the calculated Raman spectra as well as REPs. Calculations of the dynamic dielectric tensors $\varepsilon_{\alpha\beta}$ need to be repeated for the perturbed system by different atomic displacements along $\pm x$, $\pm y$ and $\pm z$ directions using the *GW*+BSE framework, so the derivatives of the dynamic dielectric tensors with respect to atomic displacements can be obtained for computing resonant Raman intensities. Here, for the equilibrium and perturbed TMDs systems, we first used the GGA exchange-correlation functional in the Perdew-Burke-Ernzerhof (PBE) [10] flavor to obtain the DFT ground states with the spin-orbit coupling (SOC) included. Note that the PBE-*GW* pseudopotentials provided by VASP were used as they are optimized for treating unoccupied states far above the Fermi level. For the equilibrium structures, both the atomic positions and in-plane lattice constants were optimized until the residual forces were below 0.001 eV/Å. For the perturbed systems, structural relaxation was not needed, but self-consistent calculations were performed. The energy cutoff was 400 eV and the k-point sampling was 24×24×1. Based on the DFT charge densities and wave functions of the equilibrium and perturbed systems, single-shot G_0W_0 calculations were subsequently carried out by VASP [11]. The energy cutoff for the response function was set at 200 eV. An appreciable number of empty bands was included in the calculations so that the total number of bands was 128. The BSE calculations were carried out on top of the

G_0W_0 quasiparticle spectrum to obtain the frequency-dependent dielectric function with the excitonic effects. BSE was solved using the Tamm-Dancoff approximation. The maximum excitation energy of included electron-hole pairs was 6 eV so that the pairs with the one-electron energy difference beyond this limit were not included in the BSE Hamiltonian. Because of the high computational cost, we used a k-point sampling of 12×12×1 for the *GW* and BSE calculations. For both positive and negative atomic displacements (Δ = 0.01 Å) in the unit cell of WS_2 and WSSe, the dynamic dielectric tensors $\varepsilon_{\alpha\beta}$ were computed at the *GW*+BSE level and then their derivatives were obtained via the finite difference scheme. Based on the phonon frequencies, phonon eigenvectors and the derivatives of dielectric tensors, Raman tensor $\tilde{R}$ and Raman intensity of any phonon mode at any laser excitation energy can be obtained, thereby yielding REPs.

We note that there is a specific relation between the Cartesian displacements and normal mode coordinates. According to the book "*Dynamical Theory of Crystal Lattices*" by Born and Huang (Part II, Chapter IV, Page 173-175, "Normal Coordinates"), the Cartesian displacement $r_l(\mu)$ of atom μ along Cartesian direction l is directly related to normal coordinates by summing over all phonon modes j :

$$r_l(\mu) = \frac{1}{\sqrt{M_\mu}} \sum_j e_l^j(\mu) Q_j \qquad \text{(S2)}$$

where $e_l^j(\mu)$ corresponds to the displacement of the μ-th atom along direction l in the j-th phonon mode (i.e., the phonon eigenvector), Q_j is the normal coordinate of phonon modes j, and M_μ is the mass of the μ-th atom. Since normal coordinates are independent of one another, the derivative of the Cartesian displacement with respect to a single specific normal coordinate Q_j is given by

$$\frac{\partial r_l(\mu)}{\partial Q_j} = \frac{e_l^j(\mu)}{\sqrt{M_\mu}} \qquad \text{(S3)}$$

Raman tensor of a phonon mode j is proportional to the derivative of the electronic polarizability tensor $\chi_{\alpha\beta}$ over the phonon normal mode coordinate Q_j:

$$\tilde{R}_{\alpha\beta}(j) \propto \frac{\partial \chi_{\alpha\beta}}{\partial Q_j}. \qquad \text{(S4)}$$

Because the electronic polarizability tensor $\chi_{\alpha\beta}$ is a macro-property that depends on the geometry of the entire system, it is a function of $3N$ distinct real-space variables:

$$\chi_{\alpha\beta} = \chi_{\alpha\beta}\big(r_x(1), r_y(1), r_z(1), \dots, r_x(\mu), r_y(\mu), r_z(\mu), \dots, r_x(N), r_y(N), r_z(N)\big), \qquad \text{(S5)}$$

where N is the number of atoms in the system. Similarly, since a normal mode is a collective movement of all atoms simultaneously, any change in the normal coordinate means changing the positions of every single atom in the system at the same time along all three directions:

$$Q_j = Q_j\big(r_x(1), r_y(1), r_z(1), \ldots, r_x(\mu), r_y(\mu), r_z(\mu), \ldots, r_x(N), r_y(N), r_z(N)\big). \quad \text{(S6)}$$

When calculating the total derivative of $\chi_{\alpha\beta}$ with respect to a single collective variable Q_j, the calculus rule for a function of multiple variables dictates that one must evaluate the partial derivative for every single coordinate, and multiply it by how that coordinate changes with respect to the target variable:

$$\frac{\partial \chi_{\alpha\beta}}{\partial Q_j} = \sum_{\mu=1}^{N} \sum_{l=x,y,z} \frac{\partial \chi_{\alpha\beta}}{\partial r_l(\mu)} \frac{\partial r_l(\mu)}{\partial Q_j} = \sum_{\mu=1}^{N} \sum_{l=x,y,z} \frac{\partial \chi_{\alpha\beta}}{\partial r_l(\mu)} \frac{e_l^j(\mu)}{\sqrt{M_\mu}} \quad \text{(S7)}$$

The expression on the right end is the same as Eq. S1. In other words, the Raman tensor of a phonon mode j is obtained via the derivatives of the polarizability (or dielectric) tensor with respect to atomic displacements **that are then multiplied by phonon eigendisplacements of the *j*-th mode**. The derivatives of the polarizability tensors with respect to the Cartesian atomic displacements are straightforward for implementation in DFT calculations.

Note S2. Correction for Resonant Si Raman scattering.

To correct for the resonant character of the 521 cm^{-1} Si Raman mode, we used the 322 cm^{-1} first-order Raman scattering mode of a CaF_2 crystal – a wide band insulator (band gap ~11eV) – that exhibits non-resonant Raman scattering, which has been widely used for this purpose.[12, 13] The relative Si Raman scattering cross-section for this mode expressed as σ_{Si}=I(521 cm^{-1} Si, E_{ph})/I(322 cm^{-1} CaF_2) has been reported by Renucci et al. [14] and has been used in many reports devoted to REPs of TMDCs, e.g., [15-18]. In our work, we used the cross-section, σ_{Si}=I(521 cm^{-1} Si, E_{ph})/I(322 cm^{-1} CaF_2), from Fig. 6 of Ref. [14] with the parameter δ=0 to correct for the resonant character of Si Raman scattering.

Note S3. Interference Correction Factor

Calculation of the interference correction factor is well established and have been described in many papers with different degree of approximation used. [15,17, 19-23] Same calculations

have been used to estimate photoluminescent (PL) and Raman scattering enhancement factors due to interference effect typically for 2D materials on SiO_2/Si substrates. These calculations are based on the Fresnel equations to calculate transmittance, t_{ij}, and reflection, r_{ij}, coefficients at the interfaces i,j of a multilayer system using complex refractive indexes, $\tilde{n}_i = n - ik$ of the layers (which in our case consists of air, TMDC monolayer (M), SiO_2, and Si labeled by the index $i = 0, 1, 2, 3$, respectively) and the corresponding phase factors as follows [16,17]:

$$t_{ij} = \frac{2\tilde{n}_i}{\tilde{n}_i + \tilde{n}_j}, \qquad r_{ij} = \frac{\tilde{n}_i - \tilde{n}_j}{\tilde{n}_i + \tilde{n}_j}, \qquad \beta_x = 2\pi\tilde{n}_1 x/\lambda, \qquad \beta_j = 2\pi\tilde{n}_j d_j/\lambda, \tag{S8}$$

where the normal incidence case is considered, and the β_x , β_j are phase changes in the case of a variable depth x and pass through complete layers with the thickness, d_j, respectively.

Here, we consider correction factor estimation for the Raman Excitation Profile (REP) of WS_2 monolayer, the same procedure is applied to calculate the correction factors for WSe_2, WSSe, and $WS_{2(1\text{-}x)}Se_{2x}$ intermediates by using their corresponding refractive indexes as described in the main text. To estimate the correction factor for the WS_2 REP, we used the following refractive indexes: n_0=1 (air); $\tilde{n}_1(\lambda)$ from the work of Jung et al., [24] (WS_2, Fig. S1a); n_2=1.461(SiO_2); $\tilde{n}_3$ (λ) from the paper of Schinke et al., [25] (Si, Fig. S1b). Note that different $\tilde{n}_1(\lambda)$ and $\tilde{n}_3(\lambda)$ should be used for excitation and scattering due to difference in their wavelengths.

The output Raman scattering intensities from the WS_2 monolayer on SiO_2/Si substrate and from Si are given by [16,17]

$$f_{WS2} = \int_0^{d1} |F_{exc}(x)F_{sc}(x)|^2 dx, \tag{S9}$$

$$f_{Si} = \int_0^{zR} |G_{exc}(x)G_{sc}(y)|^2 dy, \tag{S10}$$

where $F_{exc}(x)$ and $F_{sc}(x)$ are electric field amplitudes for the excitation and scattering at a depth x in WS_2, and similarly, $G_{exc}(x)$ and $G_{sc}(y)$ are electric field amplitudes for the excitation and scattering at a depth y in Si, d_1=0.64 nm, Z_R is Rayleigh distance defined as $Z_R(\lambda) = \frac{\pi w_0^2 n_{Si}(\lambda)}{\lambda}$, ($w_0$ is the excitation beam spot radius in focus) [17].

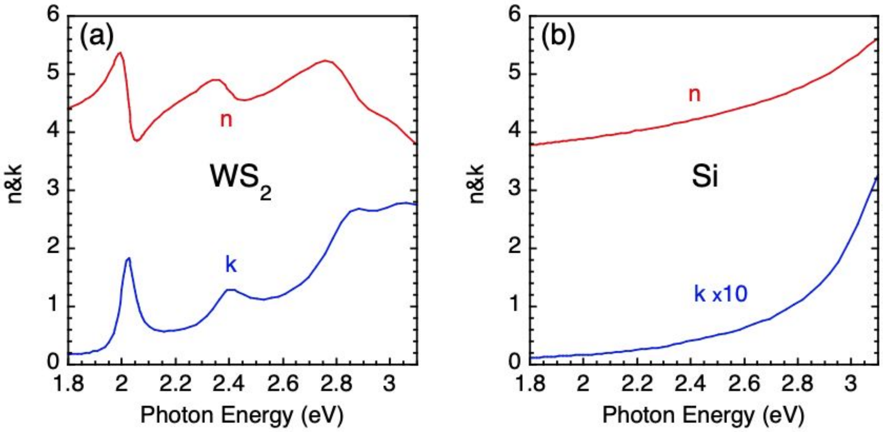

Figures S1a, b. Refractive indexes of a WS_2 monolayer (a) [24] and Si (b) [25] used to estimate correction factor for the 418 cm^{-1} A_1' mode REP of WS_2 monolayer on a SiO_2/Si substrate.

The electrical field amplitudes: $F_{exc}(x)$ and $F_{sc}(x)$; $G_{exc}(x)$ and $G_{sc}(y)$ can be calculated using Eqs. (S8-S10), which has been reported in many publications. [15,17,19] Here, using the corresponding expressions from Ref. [15], we calculated the f_{WS2} and f_{Si} for the SiO_2 layer thickness $d_2 =$ 300 nm used in our experiment (Figs. S2a, b). The final correction factor, $f(E_{ph}) = f_{WS2}(E_{ph})/f_{WS2}(E_{ph})$, ($E_{ph}$ is the photon energy) is plotted in Fig. S2c. Similar calculations were conducted for WSe_2 (with $\tilde{n}_1(\lambda)$ taken from Ref. [24]) and Janus WSSe

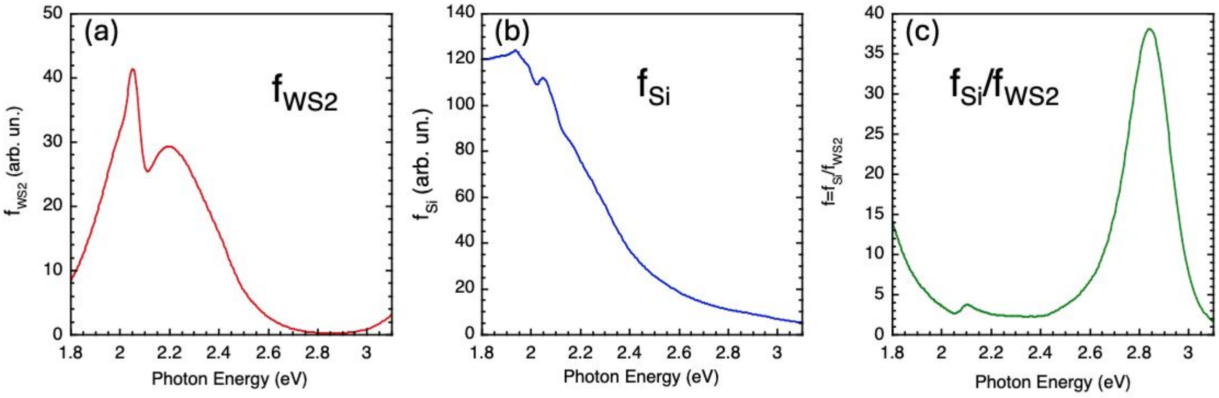

Figures S2a, b, c. Functions $f_{WS2}(E_{ph})$ and $f_{Si}(E_{ph})$ calculated using MATLAB for the 418 cm^{-1} A_1' mode of WS_2 monolayer (a) and the 521cm^{-1} mode of Si (b). The following parameters are used in the calculations:
$d_1 =$ 0.64 nm, $d_2 =$ 300 nm, λ =400 – 700 nm, $n_0 = 1$, n_2=1.461(SiO_2), $\tilde{n}_1(E_{ph})$ and $\tilde{n}_3(E_{ph})$ as plotted in Figs. S1a and S1b, respectively. (c) The final interference correction factor, $f(E_{ph})$, is defined as [illegible]

monolayers as well as for Janus intermediates. The refractive indices of Janus WSSe monolayers

and their fractional intermediates were calculated using averaging of the known indexes $\tilde{n}_{\mathrm{WS2}}(E_{ph})$ and $\tilde{n}_{\mathrm{WSe2}}(E_{ph})$ as $\tilde{n}_{\mathrm{x}} = (1-x)\tilde{n}_{\mathrm{WS2}}(E_{ph}) + \mathrm{x}\tilde{n}_{\mathrm{WSe2}}(E_{ph})$, where x is the conversion fraction and $x = 0.5$ corresponds to a Janus WSSe monolayer and E_{ph} is the photon energy.

Figure S3.

References

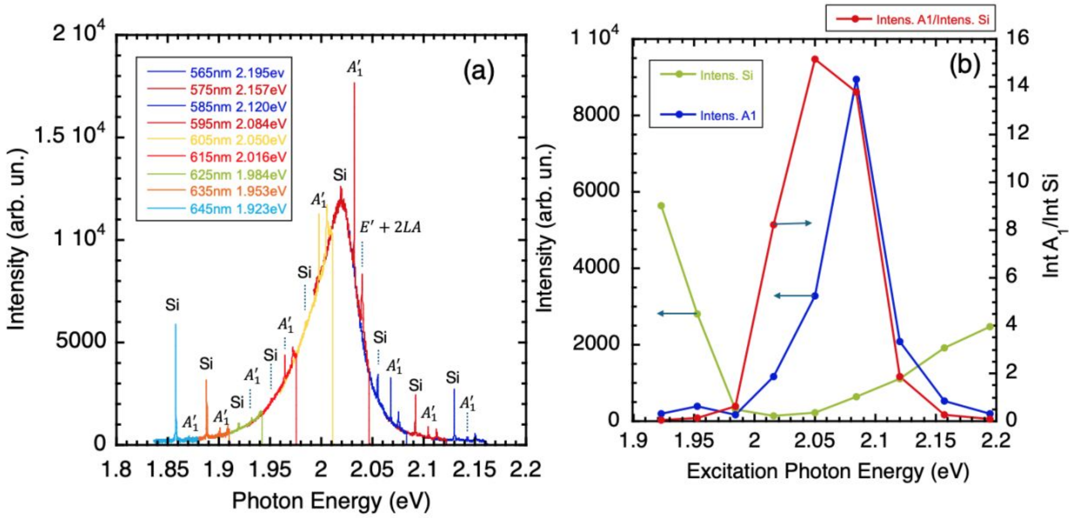

Figures S3a, b. **(a)** PL spectrum of WS_2 monolayer obtained by patching the overlapping high-resolution fractional parts with superimposed Raman lines measured at different photon energies listed in the insert. **(b)** Intensities of 521 cm^{-1} Si, 418 cm^{-1} WS_2 monolayer lines, and their ratio, which show that for the excitation photon energies in the region of strong X_A absorption the intensities of the Si and A_1' WS_2 lines are very small that can result in large errors in the A_1' /Si intensity ratio.

1. G. Kresse, J. Furthmuller, Efficiency of *ab-initio* total energy calculations for metals and semiconductors using a plane-wave basis set, *Comput. Mater. Sci.* **6**, 15-50 (1996).

2. A. Togo, F. Oba, I. Tanaka, First-principles calculations of the ferroelastic transition between rutile-type and $CaCl_2$-type SiO_2 at high pressures, *Phys. Rev. B* **78**, 134106 (2008).

3. P. Umari, A. Pasquarello, A. Dal Corso, Raman Scattering intensities in Alpha-Quartz: A First-Principles Investigation, *Phys. Rev. B* **63** 094305 (2001).

4. X. Kong, P. Ganesh, L. Liang, First-principles study of the magneto-Raman effect in van der Waals layered magnets, *npj 2D Materials and Applications* **8**:82 (2024).

5. M. Ceriotti, F. Pietrucci, M. Bernasconi, *Ab initio* Study of the Vibrational Properties of Crystalline TeO_2: The Alpha, Beta, and Gamma Phases, *Phys. Rev. B* **73**, 104304 (2006).

6. L. B. Liang, V. Meunier, First-Principles Raman Spectra of MoS_2, WS_2 and their Heterostructures. *Nanoscale* **6**, 5394-5401 (2014).

7. Nannan Mao, Shengyang Huang, Luiz Gustavo Piment Martins, Hugen Yan, Xi Ling, Liangbo Liang, Jing Kong, William A. Tisdale, Vibrational Fermi Resonance in Atomically Thin Black Phosphorus *Nano Letters* **24**, 12582-12589 (2024).

8. E. del Corro, A. Botello-Méndez, Y. Gillet, A. L. Elias, H. Terrones, S. Feng, C. Fantini, Daniel Rhodes, N. Pradhan, L. Balicas, X. Gonze, J.-C. Charlier, M. Terrones, M. A. Pimenta, Atypical Exciton−Phonon Interactions in WS_2 and WSe_2 Monolayers Revealed by Resonance Raman Spectroscopy,*Nano Lett.* **16**, 2363−2368 (2016).

9. Henrique P.C. Miranda, Sven Reichardt, GuillaumeFroehlicher, Alejandro Molina-Sańchez, Steṕhane Berciaud, and Ludger Wirtz, Quantum Interference Effects in Resonant Raman Spectroscopy of Single- and Triple-Layer $MoTe_2$ from First-Principles, *Nano Lett.* **17**, 2381−2388 (2017).

10. John P. Perdew, Kieron Burke, Matthias Ernzerhof, Generalized Gradient Approximation Made Simple, *Phys. Rev. Lett.* **77**, 3865–3868 (1996).

11. Hannu-Pekka Komsa, Arkady V. Krasheninnikov, Effects of confinement and environment on the electronic structure and exciton binding energy of MoS_2 from first principles, *Phys. Rev. B* **86**, 241201(R) (2012).

12. M. Grimsditch, M. Cardona, J. M. Calleja, F. Meseguer, Resonance in the Raman Scattering of CaF_2, SrF_2, BaF_2 and Diamond, *J. Raman Spectroscopy* **10**, 77-81 (1981).

13. P. Klar, E. Lidorikis, A. Eckmann, I. A. Verzhbitskiy, A. C. Ferrari, C. Casiraghi, Raman scattering efficiency of graphene, *Phys. Rev. B* **87**, 205435 (2013).

14. J. B. Renucci, R. N. Tyte, and M. Cardona, Resonant Raman scattering in silicon, *Phys. Rev. B* **11**, 3885 (1975).

15. Bruno R. Carvalho, Leandro M. Malard, Juliana M. Alves, Cristiano Fantini, and Marcos A. Pimenta, Symmetry-Dependent Exciton-Phonon Coupling in 2D and Bulk MoS_2 Observed by Resonance Raman Scattering, *Phys. Rev. Lett.* **114**, 136403 (2015).

16. P. Soubelet, A. E. Bruchhausen, A. Fainstein, K. Nogajewski, and C. Faugeras, Resonance effects in the Raman scattering of monolayer and few-layer $MoSe_2$, *Phys. Rev. B* **93**, 155407 (2016).

17. Amber McCreary, Jeffrey R. Simpson, Yuanxi Wang, Daniel Rhodes, Kazunori Fujisawa, Luis Balicas, Madan Dubey, Vincent H. Crespi, Mauricio Terrones, Angela R. Hight Walker, Intricate Resonant Raman Response in Anisotropic ReS_2,*Nano Lett*. **17**, 5897−5907 (2017).

18. Jae-Ung Lee, Jaesung Park, Young-Woo Son, Hyeonsik Cheong, Anomalous excitonic resonance Raman effects in few-layered MoS_2, *Nanoscale* **7**, 3229 (2015).

19. Hui Zhang, Yi Wan, Yaoguang Ma, Wei Wang, Yilun Wang, Lun Dai, Interference effect on optical signals of monolayer MoS_2, *Appl. Phys. Lett.* **107**, 101904 (2015).

20. Song-Lin Li, Hisao Miyazaki, Haisheng Song, Hiromi Kuramochi, Shu Nakaharai, and Kazuhito Tsukagoshi, Quantitative Raman Spectrum and Reliable Thickness Identification for Atomic Layers on Insulating Substrates, *ACS Nano* **6**, 7381-7388 (2012).

21. Y. Y. Wang, Z. H. Ni, Z. X. Shen, H. M. Wang, Y. H. Wu, Interference enhancement of Raman signal of graphene, *Appl. Phys. Lett.* **92**, 043121 (2008).

22. Duhee Yoon, Hyerim Moon, Young-Woo Son, Jin Sik Choi, Bae Ho Park, Young Hun Cha, Young Dong Kim, and Hyeonsik Cheong, Interference effect on Raman spectrum of graphene on SiO_2/Si, *Phys. Rev. B* **80**, 125422 (2009).

23. Nannan Mao, Xingzhi Wang, Yuxuan Lin, Bobby G. Sumpter, Qingqing Ji, Tomás Palacios, Shengxi Huang, Vincent Meunier, Mildred S. Dresselhaus, William A. Tisdale, Liangbo Liang, Xi Ling, and Jing Kong, Direct Observation of Symmetry-Dependent Electron−Phonon Coupling in Black Phosphorus, *J. Am. Chem. Soc.*, 141, 18994−19001 (2019).

24. Gwang-Hun Jung, SeokJae Yoo and Q-Han Park, Measuring the optical permittivity of two-dimensional materials without a priori knowledge of electronic transitions, *Nanophotonics* **8** 263–270 (2019).

25. Carsten Schinke, P. Christian Peest, Jan Schmidt, Rolf Brendel, Karsten Bothe, Malte R. Vogt, Ingo Kröger, Stefan Winter, Alfred Schirmacher, Siew Lim, Hieu T. Nguyen, Daniel MacDonald, Uncertainty analysis for the coefficient of band-to-band absorption of crystalline silicon, *AIP Advances* **5**, 067168 (2015).
